# Comorbidity patterns associated with severe COVID-19 outcomes: A cohort study based on the UK Biobank

**DOI:** 10.1371/journal.pone.0329701

**Published:** 2025-08-22

**Authors:** Jian Zhang, Can Hou, Wenwen Chen, Yao Hu, Shishi Xu, Haowen Liu, Yao Yang, Unnur A. Valdimarsdóttir, Fang Fang, Huan Song

**Affiliations:** 1 Mental Health Center and West China Biomedical Big Data Center, West China Hospital, Sichuan University, Chengdu, China; 2 Med-X Center for Informatics, Sichuan University, Chengdu, China; 3 West China Biomedical Big Data Center, West China Hospital, Sichuan University, Chengdu, China; 4 Division of Endocrinology and Metabolism, West China Hospital, Sichuan University, Chengdu, China; 5 Center of Public Health Sciences, Faculty of Medicine, University of Iceland, Reykjavík, Iceland; 6 Institute of Environmental Medicine, Karolinska Institute, Stockholm, Sweden; 7 Department of Epidemiology, Harvard T H Chan School of Public Health, Boston, Massachusetts, United States of America; University of Oxford, UNITED KINGDOM OF GREAT BRITAIN AND NORTHERN IRELAND

## Abstract

**Background:**

Pre-existing comorbidities are linked to increased risk of severe COVID-19, but comprehensive assessments of comorbidity patterns remain limited.

**Methods:**

We used network analysis to identify pre-existing comorbidity modules (i.e., groups of diseases more densely interconnected with each other than with other diseases in the comorbidity network) in a cohort of 420,920 individuals from the UK Biobank who were in England. We defined cases requiring hospitalization or who died of COVID-19 as “severe COVID-19”. Logistic regression was used to examine associations between comorbidity modules and severe COVID-19, and a module-based comorbidity index was developed to predict severe COVID-19, compared with existing indices.

**Results:**

Comorbidity network analysis identified 190 disease pairs with confirmed comorbidity associations, which were further divided into seven comorbidity modules. Among the 30,914 individuals diagnosed with COVID-19, 3,970 were identified as severe cases (median age of 73.6 years, 58.77% being male). Six of seven identified modules showed statistically significant associations with severe COVID-19, especially modules related to circulatory and respiratory diseases (odds ratio = 1.67 [95% confidence interval 1.54–1.81]) and age-related eye diseases (1.39 [1.27–1.52]). Associations did not differ by sex, age or vaccination status but were generally stronger during the first wave of COVID-19 pandemic (i.e., 31^st^ January-1^st^ October, 2020). Our newly developed module-based comorbidity index showed better performance in predicting severe COVID-19 (AUC = 0.779) compared to the existing Charlson Comorbidity Index (0.714) and the 16-comorbidity index (0.714).

**Conclusions:**

Our study demonstrated that pre-existing comorbidity modules, particularly modules related to circulatory and respiratory diseases and age-related eye diseases, were associated with severe COVID-19. Moreover, the module-based comorbidity index provides better prediction of severe COVID-19 than existing prediction indices.

## Introduction

As of November 2024, the global coronavirus disease 2019 (COVID-19) pandemic has affected 10% of the world’s population, resulting in approximately 137 million hospitalized cases and nearly seven million deaths [1]. Most people experience mild symptoms and recover within a few weeks, whilst others suffer severe or even life-threatening conditions which require intensive medical interventions and might lead to serious and prolonged complications [2]. Therefore, to reduce the burden of healthcare system and diminish the impact of such pandemic on society, it is important to identify individuals at high risk of progressing to severe COVID-19 (i.e., hospitalization or death due to COVID-19) and offer a basis for implementing early risk prediction.

As the pandemic continues to evolve, a considerable research effort has been devoted to characterizing risk factors potentially associated with severe COVID-19. Besides demographic and lifestyle factors such as advanced age, high body mass index (BMI) [3], and current smoking, a significant proportion of severe COVID-19 has been attributed to pre-existing comorbidities [4]. Indeed, previous studies have found that comorbidities, such as hypertension [5], diabetes mellitus [6], cardiovascular disease [7], chronic obstructive pulmonary disease [8], and chronic kidney disease [9] are associated with increased risk of hospitalization or death due to COVID-19. However, findings on other major comorbidities such as psychiatric disorders and autoimmune diseases are inconsistent or still lacking [10,11]. Instead of focusing on single diseases, recent efforts focused on diseases networks by partitioning co-occurring diseases into distinct modules with potentially shared underlying biological processes, or a shared genetic basis [12,13]. Demonstrating concurrent diseases through networks may provide a more comprehensive understanding of the links between pre-existing comorbidities and severe COVID-19, with possible explanations on the underlying biological mechanisms.

In the present study, leveraging extensive information on healthcare records and COVID-19 status in the UK Biobank, we aimed to identify pre-existing comorbidity modules in a large sample of study participants and elucidate their impact on the occurrence of severe COVID-19. Further, based on the importance of each comorbidity in the corresponding module and the impact of the module on the risk of severe COVID-19, we developed a module-based comorbidity index for predicting severe COVID-19. Together, findings of the present study have potential to advance our understanding of mechanisms underlying disease progression of COVID-19 and provide a basis for developing medical management for individuals affected by various COVID-19 variants or other contagious diseases in future pandemics of similar kind.

## Method

### Study design

The present study was based on the UK Biobank, a large community-based cohort that enrolled over 500,000 individuals aged 40−69 years across the UK between 2006 and 2010 [14]. This cohort study is described in detail elsewhere [15]. The UK Biobank has released primary care and COVID-19 test data for ~92% of participants in England to support COVID-19 research. Primary care data come from two major GP systems, and COVID-19 test results (RT-PCR) are sourced from Public Health England’s surveillance system.

Among the 502,507 participants of UK Biobank, we included 445,757 individuals after exclusion of participants who withdrew their informed consent (n = 101, S1 Fig) and those who were registered outside of England (n = 56,649) as there are no complete primary care and COVID-19 test data for participants in Scotland and Wales. We further excluded 24,837 individuals who had died or were lost to follow-up on 31^st^ January 2020 (i.e., the date of the first reported COVID-19 case in the UK), leaving 420,920 participants in the analytical cohort for the identification of comorbidity modules. Among these, 30,914 individuals were identified as COVID-19 cases, according to COVID-19 diagnostic tests, diagnoses in the primary care (codes listed in S1 Table) or inpatient hospital data (International Classification of Diseases 10^th^ edition [ICD-10]: U07·1 or U07·2), and underlying cause of death in the mortality data (ICD-10: U07.1 or U07.2). Follow-up for severe COVID-19 among all cases started from their date of diagnosis until hospitalization or death within 30 days post-diagnosis or the end of the study (November 30, 2021), whichever occurred first. The incidence rate of COVID-19 was comparable between the analytical cohort and the entire UK as reported by the Office for National Statistics [16] (see S2 Table), suggesting that the majority of COVID-19 cases were captured in this analysis.

The UK Biobank study has received full ethical approval from the NHS National Research Ethics Service (16/NW/0274), and all participants provided written informed consent before data collection. The present study was approved by the biomedical research ethics committee of West China Hospital (2020.661).

#### Ascertainment of pre-existing comorbidities.

We used the term ‘comorbidity’ broadly to encompass a spectrum of medical conditions, including both chronic and acute illnesses, that were diagnosed before COVID-19 [17,18]. Since we focused on diseases that are common in the general population with substantial impact on healthcare systems, we restricted our analyses to a total of 20 communicable and 95 non-communicable diseases listed in the Global Burden of Disease (GBD) Study 2019 [19]. All diagnoses (i.e., main and secondary) in the primary care and inpatient hospital data before 31^st^ January 2020 were used for disease ascertainment, according to corresponding ICD-10 codes (see S3 Table). Specifically, as primary care diagnoses are coded using SNOMED-CT and Read V3 codes, these were converted to the latest release of cross-maps provided by NHS digital ICD-10 codes [20,21], as used for inpatient hospital data.

#### Ascertainment of severe COVID-19.

Severe COVID-19 cases were defined as (i) those admitted to hospital or who died with this diagnosis, or (ii) those admitted to hospital with or without a COVID-19 diagnosis, or who died, within 30 days of a positive COVID-19 diagnostic test or a COVID-19 diagnosis recorded in the primary care data. For subsequent hospital admissions or deaths lacking a COVID-19 diagnosis, inclusion was restricted to those potentially related to COVID-19, excluding underlying causes of death or primary diagnosis codes related to pregnancy, perinatal conditions, symptoms, or signs, as well as external causes of morbidity and mortality (refer to S4 Table).

#### Covariates.

Sociodemographic and lifestyle factors, including date of birth, sex, annual household income, and smoking and drinking status, were collected using touchscreen questionnaires at recruitment. The Townsend deprivation index (TDI), a widely used measure of population-level deprivation [22], was assigned to each participant based on the postal codes provided at recruitment. Additionally, we calculated body mass index (BMI) using their height and weight measurements. We extracted COVID-19 vaccination information for each participant from primary care data, using a list of predefined SNOMED-CT and Read V3 codes (see S5 Table).

### Statistical analysis

#### Comorbidity network analysis for identification of comorbidity modules.

The comorbidity network analysis was constructed to investigate the diversity of pre-existing disease patterns prior to the diagnosis of COVID-19. We followed previously described steps of comorbidity network analysis to identify comorbidity modules (i.e., groups of diseases more densely interconnected with each other than with other diseases in the comorbidity network) [18]. A detailed description of the analysis steps is available in S1 File. Specifically, disease pairs with sufficient prevalence (i.e., co-occurrence in at least 1% of the study population) and comorbidity strength (measured by Pearson’s correlation and relative risk of a disease pair in the same individual) were pre-selected and subsequently verified using logistic regression, controlling for potential confounders. The selected disease pairs were used to construct a comorbidity network, and comorbidity modules within the network (i.e., clusters of highly interconnected comorbidities) were identified using the Louvain community detection algorithm [23].

#### Association analyses.

Logistic regression was used to investigate the associations between pre-existing comorbidity modules and risk of severe COVID-19. For each comorbidity module identified, we first calculated the odds ratio (OR) of severe COVID-19 in relation to being diagnosed with any disease in the module, adjusting for age, sex, annual household income, TDI, BMI, smoking status, drinking status, and disease status of other modules (i.e., being diagnosed with any disease in the corresponding module). Additionally, we estimated ORs for being diagnosed with different numbers of comorbidities within a module (i.e., 1, 2, and 3+). Finally, we calculated the OR of severe COVID-19 in relation to being diagnosed for each individual disease of the comorbidity module.

We conducted sub-analyses for males and females separately. To assess the effect of age, we also conducted sub-analyses for individuals with age < 66 and those≥66 years (i.e., median age) separately. To examine age-related differences, participants were grouped by the median age (66 years) into <66 and ≥66 years. To investigate the effect of vaccination on the association between comorbidity modules and severe COVID-19, we conducted separate analyses for COVID-19 cases with and without vaccination at the time of diagnosis. To further investigate the influence of vaccination status on the observed associations, we stratified COVID-19 cases with vaccination based on the time from the last vaccination does to the COVID-19 diagnosis (i.e., < 6 months or ≥6 months). Finally, we repeated the analyses for COVID-19 cases diagnosed during the first wave (before 1^st^ October, 2020) and second wave (after 1^st^ October, 2020) of the COVID-19 pandemic in the UK separately, to investigate the influence of different viral strains on the results [24,25]. Difference in ORs was assessed by introducing an interaction term to the logistic regression.

#### Module-based comorbidity index.

Based on the identified comorbidity modules and their association with severe COVID-19, we developed a module-based comorbidity index to predict the risk of severe COVID-19. A detailed description of the analysis steps is available in Supplementary Methods. The index included 51 diseases, with weight of each disease calculated as the product of the disease’s importance in the corresponding module and the OR of the association between the corresponding module and severe COVID-19. The importance of each disease in a module was estimated using a previously proposed importance ranking method for complex networks [26]. We also developed six simplified module-based comorbidity indices by including the top 15, 20, 25, 30, 35, or 40 diseases with the highest weight, among the 51 diseases, to understand if it is possible to reduce the information needed for calculating the comorbidity score.

To evaluate the performance of the module-based comorbidity index in predicting severe COVID-19, we compared its performance with two existing indices, the Charlson Comorbidity Index (CCI) [27] and a 16-comorbidity based index [28]. We used both Support Vector Machine (SVM) and Extreme Gradient Boosting (XGBoost) models that incorporated age and sex as additional covariates in this comparison. We randomly divided the dataset into training and test sets in a 9:1 ratio for index development and evaluation, respectively. The area under the receiving operating characteristic curve (AUC) was chosen as the primary evaluation metric, and differences in AUC between indices were compared using the DeLong test [29]. Additionally, we considered the Akaike information criterion (AIC) [30] and Bayesian information criterion (BIC) [31] as secondary evaluation metrics. The detailed workflow is depicted in S2 Fig.

All the analyses were carried out using R (version 3·6·2, R Foundation for Statistical Computing, Vienna, Austria) and Python 3·8 (Python Software Foundation Delaware, USA), with two-sided *P*-value<0·05 as statistically significant.

## Results

We included a total of 420,920 participants as our study population for comorbidity network analysis. The median age at recruitment was 69·30 years, and 54·31% of the participants were females. Among the 115 comorbidities studied, the most prevalent non-communicable diseases included bacterial skin diseases, low back pain, and anxiety disorders, while the most prevalent communicable diseases included upper respiratory infections and lower respiratory infections. A total of 30,914 individuals with COVID-19 were identified in the study population, of which 3,970 were classified as severe COVID-19 (S1 Fig and S6 Table). Compared to mild COVID-19 cases, severe COVID-19 cases were generally older (median age 73·6 vs 64·6*, P *< .001) and more likely to be male (58·77% vs. 45·18%*, P *< .001), obese (41·61% vs. 27·75% for BMI ≥ 29·9*, P *< .001), and current smokers (41·86% vs. 34·22%*, P *< .001) but less likely to have a higher household income (10·88% vs. 23·49% for household income ≥52,000£*, P *< .001) and be current drinkers (84·89% vs. 91·50%*, P *< .001) (Table 1).

**Table 1:** Baseline characteristics of the study participants with COVID-19.

Characteristics	Mild COVID-19 cases (N = 26,944)	Severe COVID-19 cases (N = 3,970)	*P-values*
Age at recruitment, years	64·6 (58·2-72·7)	73·6 (66·30-78·0)	<.001
Sex			<.001
*Female*	14,882 (55·23%)	1,637 (41·23%)	
*Male*	12,172 (45·18%)	2,333 (58·77%)	
Townsend deprivation index			<.001
*<−3·64*	5,869 (21·78%)	666 (16·78%)	
*−3·64 to −2·14*	6,301 (23·39%)	776 (19·55%)	
*−2·14 to 0·55*	6,965 (25·85%)	974 (24·53%)	
*≥ 0·55*	7,778 (28·87%)	1,550 (39·04%)	
*Unknown*	31 (0·12%)	< 5(0·10%)	
Household income, ₤			<.001
*< 18000*	4,718 (17·51%)	1,266 (31·89%)	
*18000–52000*	12,244 (45·44%)	1,400 (35·26%)	
*≥ 52000*	6,330 (23·49%)	432 (10·88%)	
*Unknown*	3,652 (13·55%)	872 (21·96%)	
Body mass index, kg/m2			<.001
*<24·1*	5,864 (21·76%)	509 (12·82%)	
*24·1 to 29·9*	13,437 (49·87%)	1,747 (44·01%)	
*≥ 29·9*	7,476 (27·75%)	1,652 (41·61%)	
*Unknown*	167 (0·62%)	62 (1·57%)	
Smoking status			<.001
*Never smoker*	14,751 (54·75%)	1,713 (43·15%)	
*Current smoker*	9,219 (34·22%)	1,662 (41·86%)	
*Former smoker*	2,827 (10·49%)	546 (13·75%)	
*Unknown*	147 (0·55%)	49 (1·23%)	
Drinking status			<.001
*Never drinker*	1,338 (4·97%)	312 (7·86%)	
*Former drinker*	867 (3·22%)	261 (6·57%)	
*Current drinker*	24,655 (91·50%)	3,370 (84·89%)	
*Unknown*	84 (0·31%)	27 (0·68%)	

The values are reported as median (lower quantile-upper quantile) for continuous variables and number (%) for categorical variables at the time of enrollment into the UK Biobank.

### Comorbidity modules

**Fig 1:**
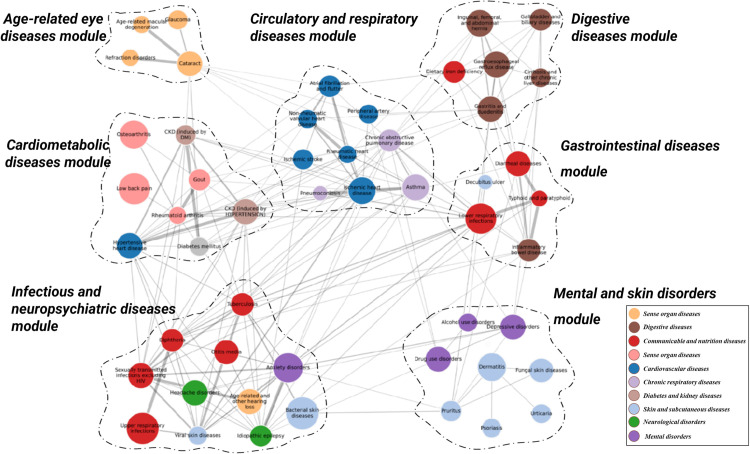
Comorbidity networks for pre-pandemic diseases. Each node represents a disease, while the size and color of the node indicate the prevalence and category of the corresponding disease, respectively. The width of the link represents the strength of the comorbidity association, measured by odds ratio from logistic regression. The network is partitioned into seven modules using Louvain algorithm, and nodes belonging to the same module are grouped together and separated from other nodes using dashed lines.

The comorbidity network analysis identified 190 disease pairs with confirmed comorbidity associations, which were further divided into seven comorbidity modules (Fig 1). According to the predominant diseases in the module, they were named as age-related eye diseases module, cardiometabolic diseases module, infectious and neuropsychiatric diseases module, circulatory and respiratory diseases module, gastrointestinal diseases module, digestive diseases module, and mental and skin disorders module, respectively.

### Main analyses

**Fig 2:**
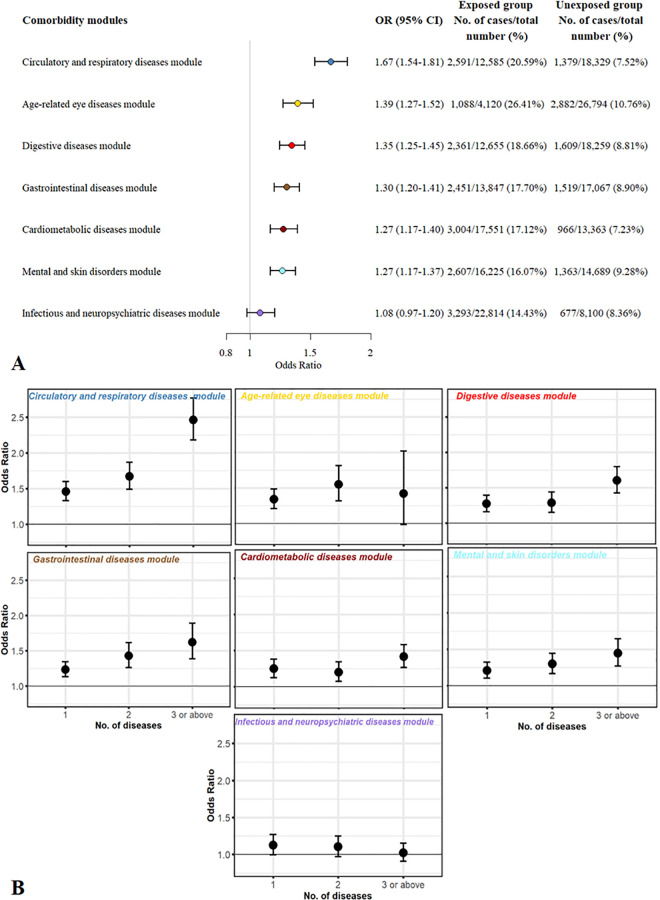
Association between pre-existing comorbidity modules and severe COVID-19. A, association between individual comorbidity modules and risk of severe COVID-19; B, association between numbers of diagnosed diseases within a module and risk of severe COVID-19. OR and the corresponding 95% CI were derived from logistic regression, adjusted for age, sex, Townsend deprivation index, annual household income, BMI, smoking status, drinking status, and disease status of other modules.

**Fig 3:**
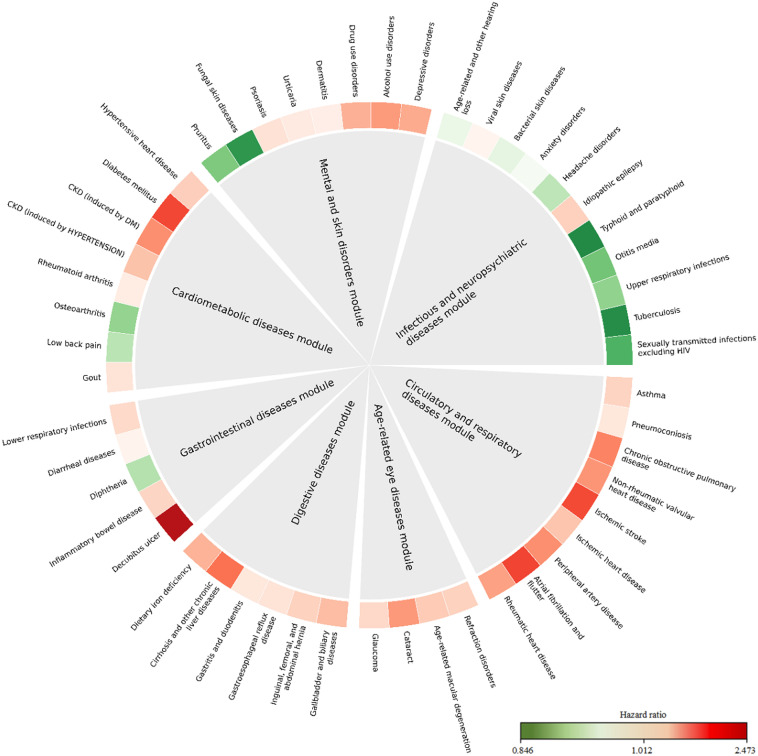
The association between individual diseases in each comorbidity module and risk of severe COVID-19. OR and the corresponding 95% CI were derived from the logistic regression, adjusted for age, sex, Townsend deprivation index, household income, BMI, smoking, and alcohol drinking status. The red color indicates positive association (i.e., OR>1) and the green color indicates negative association (i.e., OR<1). The degree of color represents the magnitude of the corresponding association. Detailed results are shown in S5 Table.

As shown in Fig 2A, all six modules, except the infectious and neuropsychiatric diseases module, demonstrated statistically significant associations with severe COVID-19. The strongest association was found for the circulatory and respiratory diseases module (OR: 1·67, 95% CI: 1·54–1·81), followed by age-related eye diseases module (OR: 1·39, 95% CI: 1·27–1·52), digestive diseases module (OR: 1·35, 95%CI: 1·25–1·45), gastrointestinal diseases module (OR: 1·30, 95%CI: 1·20–1·41), cardiometabolic diseases module (OR: 1·27, 95%CI: 1·17–1·40), and mental and skin disorders module (OR: 1·27, 95%CI: 1·17–1·37). The magnitude of the association for each individual disease was generally similar to that of the comorbidity module it belonged to, with the highest ORs observed for decubitus ulcer, atrial fibrillation and flutter, and diabetes mellitus (Fig 3 and S7 Table). Notably, 14 diseases were found to be associated with a reduced risk of severe COVID-19, mainly in the infectious and neuropsychiatric diseases module (Fig 3). Further analyses by numbers of diagnosed comorbidities in each module revealed that out of the seven modules, only two exhibited a distinct dose-response relationship between increasing number of comorbidities in the same module and increasing risk of severe COVID-19 (Fig 2B).

The sub-analyses revealed that the results were largely similar for most comorbidity modules between males and females, although females had stronger associations for modules related to gastrointestinal diseases (OR for females vs. males: 1·43 vs. 1·21, *P*-value for interaction = .048), and mental and skin disorders (1·39 vs. 1·18, *P*-value = .049; S3 Fig). Similarly, age-stratified analyses indicated generally consistent associations across age groups, except for the circulatory and respiratory disease module, which showed a stronger association in the older group (OR for ≥66 vs. < 66 years: 1.90 vs. 1.43; P for interaction = .006; S4 Fig). Although a lower proportion of severe COVID-19 cases were noted among COVID-19 cases that were vaccinated at the time of diagnosis, the observed association was slightly stronger among vaccinated patients than the unvaccinated patients for most comorbidity modules, apart from the circulatory and respiratory diseases module (OR for vaccinated vs. unvaccinated: 1·31 vs. 1·83, *P*-value = .002) and the cardiometabolic diseases module (OR for vaccinated vs. unvaccinated: 1·11 vs. 1·38, *P*-value = .191) (S5 Fig). No clear difference in ORs was identified when stratified the time since the last vaccine dose to the COVID-19 diagnosis (S6 Fig). Finally, the stratification analyses by the wave of the COVID-19 pandemic revealed stronger associations for almost all the comorbidity modules during the first wave, compared to the second wave. The largest difference was noted for infectious and neuropsychiatric diseases module (OR for wave 1 vs. wave 2: 1·94 vs. 1·11, *P*-value < .001), followed by digestive diseases module (1·27 vs. 1·74, *P*-value = .002) (S7 Fig).

### Module-based comorbidity index

**Fig 4:**
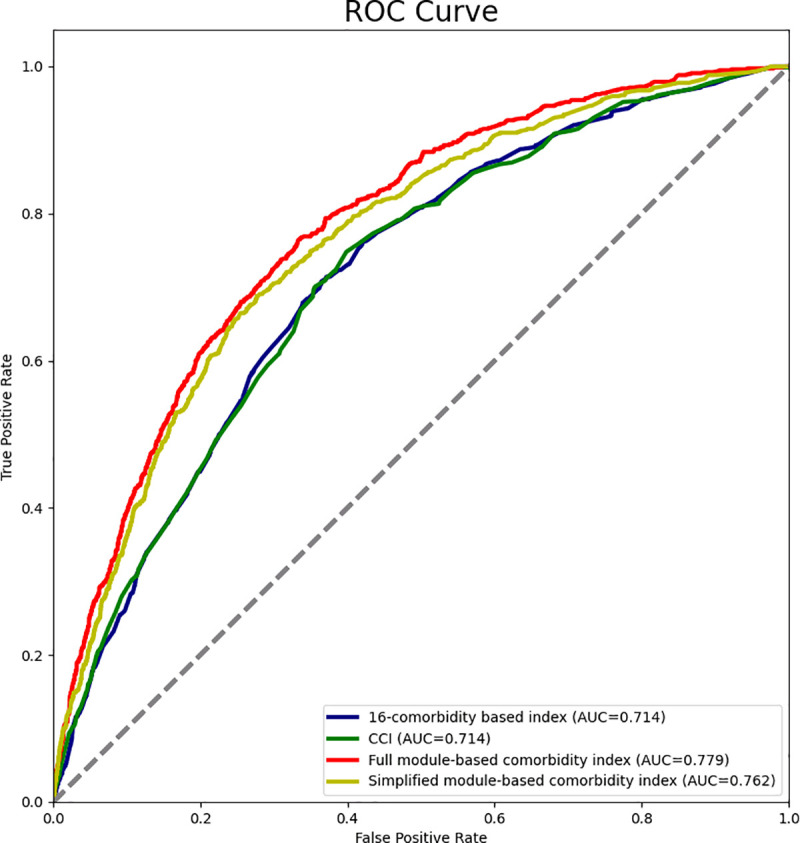
Receiver-operating characteristic curves for predicting severe COVID-19 using XGBoost model. AUC, the area under the receiving operating characteristic curve; CCI, Charlson Comorbidity Index. 16-comorbidity based index was reported in previous literature, including 16 diseases. The full module-based comorbidity index included all 51 diseases, and the simplified module-based comorbidity index included the top 15 diseases.

The weights of individual diseases in different comorbidity modules used to calculate the module-based comorbidity index are presented in S8 Table. Cataract in the age-related eye diseases module was assigned the highest weight (5·00), followed by diphtheria in the gastrointestinal diseases module (3·39) and four diseases in the circulatory and respiratory diseases module (i.e., asthma, atrial fibrillation and flutter, ischemic heart disease, and chronic obstructive pulmonary disease, with a weight of 3·14, 2·50, 2·48 and 2·25, respectively). The full module-based comorbidity index, which included all 51 diseases, achieved a statistically significantly higher AUC of 0·779 on the XGBoost model, compared to the CCI (0·714) and 16-comorbidity index (0·714), as confirmed by the DeLong test (*P* < .001) (Fig 4). Furthermore, the module-based comorbidity index also demonstrated favorable performance on the secondary evaluation metrics, as evidenced by its lower AIC and BIC values (S9 Table). Although the predictive performance of the module-based comorbidity index decreased with the number of included diseases, the simplified index, which included the top 15 diseases, still achieved satisfactory results (S9 Table). Using the SVM model, we further confirmed the superior predictive performance of the module-based comorbidity index compared to the CCI and 16-comorbidity index (S9 Table).

## Discussion

In this cohort study based on UK Biobank, we identified six distinct comorbidity modules that were statistically significantly associated with severe COVID-19, with the highest effect size observed for modules characterized by circulatory and respiratory diseases followed by age-related eye diseases. We further noted that females with comorbidity modules related to gastrointestinal diseases and mental and skin disorders were more susceptible to severe COVID-19. We further found stronger associations between majority of the comorbidity modules and risk of severe COVID-19 during first wave than second wave of the pandemic while limited attenuation by vaccination status. Finally, the new module-based comorbidity index that outperformed existing prediction indices in predicting severe COVID-19. Overall, our study provided valuable insights into the relationship between pre-existing comorbidity patterns and severe COVID-19 and has important implications for identifying high-risk individuals and personalized medical care at the time of pandemic like the COVID-19.

While no existing studies have examined the relationship between comorbidity modules and risk of severe COVID-19, our finding that six comorbidity modules are associated with the risk of severe COVID-19 aligns with finding of previous studies investigating individual or groups of diseases, including chronic obstructive pulmonary disease [32], stroke [33], diabetes [34], depression [35], inflammatory bowel disease [36], and cirrhosis [37]. Notably, the circulatory and respiratory diseases module exhibited the strongest association with severe COVID-19, corroborating several prior studies that identified respiratory diseases [38] as well as hypertension and cardiovascular diseases [39] as potent risk factors for severe COVID-19. This finding could be attributed to the high expression of angiotensin-converting enzyme 2 (ACE2) in the lung, heart, and blood vessels [40], with receptors serving as important binding sites for severe acute respiratory syndrome coronavirus 2 (SARS-CoV-2) [41]. In addition, individuals with respiratory diseases may have a heightened risk due to their anatomically small airways and impaired pulmonary function [42]. We found that age-related eye diseases module was also strongly related to severe COVID-19. This finding is supported by two recent studies where individuals with exudative age-related macular degeneration and individuals on a waiting list for cataract surgery were shown to have a higher risk of developing severe COVID-19 [43,44]. A potential explanation for this finding is that eye diseases may be an indicator of biological aging and chronic inflammation, which are also well-established risk factors for severe COVID-19. We observed strong associations between the digestive diseases module and gastrointestinal diseases module with severe COVID-19 [43,45,46]. This association might be linked to medications, including those with anticholinergic effects or impacting the gastrointestinal system [47]. Conversely, our study found that the infectious and neuropsychiatric diseases module, although common, was not statistically significantly associated with risk of severe COVID-19. This result may be possibly attributed to heightened self-protection awareness [48], and dysregulated immune responses, specifically contributing to certain components involved in anti–COVID-19 immune response [49] among individuals with these conditions.

Stratification analysis by sex revealed stronger associations for gastrointestinal diseases and mental and skin disorders in females, compared to males. This may be due to the fact that females typically possess stronger innate and adaptive immune responses than males, particularly in the presence of specific diseases (i.e., inflammatory bowel diseases and depression) [50,51]. In the case of COVID-19, this heightened immune response may also elevate the risk of cytokine imbalances, thereby increasing the likelihood of severe COVID-19 in females. Age-stratified analyses showed generally consistent associations across age groups, suggesting these links are not solely attributable to age, although modest differences in effect size were noted for individual diseases, particularly those within the circulatory and respiratory module. In the stratification analysis based on vaccination status, we observed a marginal impact of vaccination. We speculate this might be attributed to the prioritization of early vaccine recipients based on risk factors associated with severe outcomes [52]. Our findings suggest that the presence of comorbidities is equally significant for COVID-19 patients, irrespective of their vaccination status. In the stratification analysis by waves of the pandemic, we observed stronger associations between different comorbidity modules and risk of severe COVID-19 during wave 1. While this disparity might be largely attributed to delayed diagnosis (due to lack of testing for instance) and limited treatment for COVID-19 during wave 1 [25], other factors such as changes in viral strains and improved awareness and preparedness during wave 2 [24,53] could also have contributed.

The proposed module-based comorbidity index achieved superior risk prediction for severe COVID-19 when compared to existing indices like CCI and the 16-comorbidity index, across all predictive criteria. One of the major advantages of this index is its comprehensive coverage of 51 diseases, which were selected from 115 common diseases through a data-driven approach. This enables us to identify a wide range of diseases that may affect patient outcomes to include in one index for an accurate risk prediction. In comparison, CCI and the 16-comorbidity indices have a limited definition of comorbidities and exclude many common conditions [54]. Additionally, unlike existing indices that either treat diseases equally or only consider individual diseases, our index considers both the magnitude of association between a comorbidity module and severe COVID-19 and the importance for each disease for the specific module. This is under the assumption that comorbidities of a same comorbidity module typically impact health outcomes in a modular fashion, as opposed to individual diseases. Consequently, these two advantages make module-based comorbidity index a more comprehensive and accurate comorbidity index in predicting the risk of severe COVID-19, and severe outcomes of future pandemics.

The present study has several key strengths. Firstly, utilizing a comorbidity network analysis approach in the study allowed us to comprehensively assess the association between comorbidity patterns and severe COVID-19. Secondly, by leveraging enriched data on healthcare records and COVID-19 test results, with full coverage for UK Biobank participants in England, we were able to identify the majority of COVID-19 cases in the study cohort and minimize information bias. Lastly, detailed sociodemographic and lifestyle factors collected in the UK Biobank enabled us to consider several important confounders in the analysis.

However, our study also has some limitations. First, the dynamic changes over the COVID-19 pandemic, such as the evolution of virus, use of vaccine boosters and some novel medical interventions, may lead to uncertainties about the implication of our findings. However, a population-based study in the UK has found that, after the first vaccine booster, older people, those with high multimorbidity, and those with certain underlying health conditions remain at the highest risk of COVID-19-related hospitalization and death [55]. Therefore, the importance of multimorbidity to COVID-19 severity may persist. Additionally, for a broader picture that outside of the current COVID-19 challenges, our study offers a novel approach to study or capture the joint influence of comorbidities (i.e., the developed module-based comorbidity index) on a health issue, which could consequently contribute to a quicker or more comprehensive risk assessment for future epidemic, in terms of refining preventive and therapeutic approaches by pinpointing high-risk groups.

Other limitations of the current study include that external validation using datasets outside of the UK Biobank is needed to validate the module-based comorbidity index. Additionally, our definition for severe COVID-19 can be debated and may have limited the generalizability of our findings to clinical settings (e.g., identifying high risk COVID-19 patients requiring mechanical ventilation). Furthermore, the comorbidity network analysis method employed in our study has inherent limitations, and it may not fully eliminate the influence of other confounding variables on the comorbidity associations. Despite our efforts to control for environmental confounders, such as sociodemographic factors and lifestyle risk factors (i.e., BMI, drinking status, smoking status, household income, physical activity, Townsend deprivation index, household income), the influence of other comorbidities in the network was not explicitly controlled for in the model and may have contributed to observed complex comorbidity associations. Moreover, the confounding factors were collected at baseline, which may not reflect the status at the time of COVID-19 diagnosis. Although we had adjusted for multiple confounders in the analysis, residual confounding due to unmeasured factors like comorbidity severity and medication use cannot be ruled out. Lastly, it is important to note that UK Biobank participants are representative of the entire UK population or European population.

## Conclusions

Based on a community-based cohort study, we identified six disease clusters that were associated with severe COVID-19, with circulatory and respiratory diseases module showing the strongest association. Additionally, we observed that the association of gastrointestinal diseases module and mental and skin disorders module with severe COVID-19 were more pronounced for females than males. Finally, we developed a module-based comorbidity index that outperformed existing indices in predicting severe COVID-19. Our findings provide valuable insight on the relationship between pre-existing comorbidity patterns and severe COVID-19 and have important implications for identifying high-risk individuals and personalized medical care.

## Supporting information

S1 File Comorbidity network and prediction methods. (PDF)

S1 Table COVID-19 identification in primary care. (PDF)

S2 Table The theoretical and observed infection rate in the study population. (PDF)

S3 Table List of 115 GBD codes used in the current study. (PDF)

S4 Table Diagnostic codes for the identification of severe COVID-19. (PDF)

S5 Table COVID-19 vaccination identification in primary care. (PDF)

S6 Table The ICD-10 codes for the identification of severe COVID-19 case. (PDF)

S7 Table The association between each individual disease and the risk of severe COVID-19. Adjusted for age, sex, annual household income, TDI, BMI, smoking status, drinking status, and the disease status of other modules.
(PDF)

S8 Table The node importance of diseases in seven comorbidity modules. (PDF)

S9 Table Comparison of comorbidity indices in predicting risk of severe COVID-19. AIC, Akaike Information Criterion; AUC, the area under the receiver operating characteristic curve; BIC, Bayesian Information Criterion; CCI, Charlson Comorbidity Index; SVM, Support Vector Machine; XGBoost, EXtreme Gradient Boosting.16-comorbidity based index was reported in previous literature, including 16 diseases. The full module-based comorbidity index included all 51 diseases.
(PDF)

S1 Fig Flowchart of the study population selection. The first COVID-19 case was confirmed on 31 January 2020 in the UK.
(TIF)

S2 Fig Flow chart of constructing the predictive model. AIC, Akaike Information Criterion; AUC, the area under the receiver operating characteristic curve; BIC, Bayesian Information Criterion; CCI, Charlson Comorbidity Index. c16 comorbidities reported in previous literature, including coronary artery disease, heart failure, atrial fibrillation, T1DM, T2DM, hypertension, asthma, COPD, cancer, dementia, depression, anxiety, psychosis, bipolar, cognitive impairment, and stroke.
(TIF)

S3 Fig Sex-specific associations between comorbidity modules and severe COVID-19. ORs (95%CI) were derived from fully adjusted logistic models (adjusted for age, Townsend deprivation index, annual household income, BMI, smoking status, drinking status, and disease status of other modules). The interaction between comorbidity modules and sex was evaluated by including the interaction term in the logistic models. * *P* < .05.
(TIF)

S4 Fig Associations between comorbidity modules and severe COVID-19, stratified by median age. ORs (95%CI) were derived from fully adjusted logistic models (adjusted for sex, Townsend deprivation index, annual household income, BMI, smoking status, drinking status, and disease status of other modules). The interaction between comorbidity modules and sex was evaluated by including the interaction term in the logistic models. * P < .05.
(TIF)

S5 Fig Association between comorbidity modules and severe COVID-19, stratified by vaccination status at diagnosis. ORs (95%CI) were derived from fully adjusted logistic models (adjusted for age, sex, Townsend deprivation index, annual household income, BMI, smoking status, drinking status, and disease status of other modules). The interaction between comorbidity modules and vaccination status was evaluated by including the interaction term in the logistic models. * *P* < .05.
(TIF)

S6 Fig Association between comorbidity modules and severe COVID-19, stratified by time of last vaccination dose at diagnosis. ORs (95%CI) were derived from fully adjusted logistic models (adjusted for age, sex, Townsend deprivation index, annual household income, BMI, smoking status, drinking status, and disease status of other modules).
(TIF)

S7 Fig Association between comorbidity modules and severe COVID-19 stratified by different waves of the pandemic. ORs (95%CI) were derived from fully adjusted logistic models (adjusted for age, sex, Townsend deprivation index, annual household income, BMI, smoking status, drinking status, and disease status of other modules). The interaction between comorbidity modules and different waves was evaluated by including the interaction term in the logistic models. * *P* < .05.
(TIF)

## Abbreviations

ACE2: angiotensin-converting enzyme 2
AIC: akaike information criterion
AUC: the area under the receiver operating characteristic curve
BIC: Bayesian Information Criterion
BMI: high body mass index
CI: confidence interval
COPD: chronic obstructive pulmonary disease
COVID-19: coronavirus disease 2019
DIL: node importance ranking method
GBD: Global Burden of Disease
ICD-10: International Classification of Diseases 10th edition
NHS: National Health Service
OR: odds ratio
RR: relative risk
SARS-CoV-2: severe acute respiratory syndrome coronavirus 2
SVM: support vector machine
TDI: Townsend deprivation index
XGBoost: EXtreme Gradient Boosting
