# Supplementary material for: Comorbidity patterns associated with severe COVID-19 outcomes: A cohort study based on the UK Biobank
Source: PLoS One. 2025 Aug 22;20(8):e0329701. doi: 10.1371/journal.pone.0329701 (PMC12373198; doi:10.1371/journal.pone.0329701)
Supplement: S1 File — (PDF) [file pone.0329701.s001.pdf]

S1 File. Comorbidity Network and Prediction Methods.

1. Comorbidity network analysis

Step 1: The initial step involves pre-selecting disease 1 (D1) and disease 2 (D2) pairs with considerable comorbidity strength. Start by constructing all possible disease pairs using medical conditions selected from the Global Burden of Disease (GBD) codes. For each pair of diseases, create sub-cohorts by excluding individuals who have the corresponding medical condition or a history of related medical conditions. If either of the medical conditions in a disease pair is sex-specific, further restrict the sub-cohort to either males or females. Then, if the co-occurrence of these two medical conditions was experienced by at least 1.0% of the individuals in the sub-cohort, relative risk (RR) and Φ-correlation were calculated using the following formulas [1]:

$$RR_{ij} = \frac{C_{ij}N_{ij}}{C_iC_j} \tag{1}$$

$$\Phi_{ij} = \frac{C_{ij}N_{ij} - C_iC_j}{\sqrt{C_iC_j(N_{ij} - C_i)(N_{ij} - C_j)}} \tag{2}$$

Where C_{ij} is the number of individuals affected by both medical conditions, N_{ij} is the number of individuals in the sub-cohort, C_i is the number of individuals affected by D1, and C_j is the number of individuals affected by D2. For both RR and Φ-correlation measures, the significance of RR=0 and Φ=0 can be determined using a z-test. The corresponding z-score for RR and Φ-correlation can be calculated using the following formulas [1, 2]:

$$z_{ij}^{\mathrm{RR}} = \frac{\ln{(RR_{ij})}}{\sqrt{\frac{1}{C_{ij}} - \frac{1}{N_{ij}} + \frac{1}{C_iC_j/N_{ij}} - \frac{1}{N_{ij}}}} \tag{3}$$

$$z_{ij}^{\Phi} = \frac{\Phi_{ij}\sqrt{\max{(C_i, C_j)} - 2}}{\sqrt{1 - {\Phi_{ij}}^2}} \tag{4}$$

P-values were then calculated using the z-score and adjusted for the issue of multiple testing. Disease pairs with significant comorbidity strength were identified by considering a significant relative risk (RR) >1.5 and Φ-correlation >0 (i.e., q-value <0.05 for both measures). These eligible disease pairs would proceed to the next step.

Step 2: For each pre-selected disease pair, unconditional logistic regression models were used to further verify the comorbidity associations between D1 and D2 in the corresponding sub-cohort, while adjusting for household income, BMI, smoking status, drinking status, and Townsend deprivation index. D1↔D2 disease pairs with confirmed comorbidity association (i.e., OR >1.0 and q-value <0.05 in the unconditional logistic regression) were used to construct comorbidity network construction. Finally, we applied Louvain method to partition the networks into several modules with high intrinsic networks. The Louvain method is based on the following assumption: the network should have nodes (medical conditions) that are strongly interconnected. In the context of the disease comorbidity network, this assumption is quite palatable.

2. Module-based comorbidity index calculation

First, a node importance ranking method was employed to assess the significance of disease nodes within our comorbidity network. Subsequently, the weight of each disease was determined by multiplying its importance within the corresponding module by the odds ratio (OR) of the association between the respective module and severe COVID-19. The comorbidity index of an individual is defined as the sum of the disease weights associated with each disease they suffer from. The formulas for the calculations are as follows [3]:

$$L_{vi} = k_i + \sum_{v_j \in \Gamma_j} \frac{(k_i - p - 1)(k_j - p - 1)}{\frac{p}{2} + 1} \cdot \frac{k_i - 1}{k_i + k_j - 2} \tag{5}$$

$$Comorbidity\ index_m = \sum_{m \in G} (\beta_m \cdot \sum_{i \in m} \frac{L_{vi}}{S_m}) \tag{6}$$

$$S_m = \sum_{i \in m} (L_{vi}) \tag{7}$$

Where Γ_j is the set of neighbors for v_i, k_i and k_j are the degrees of v_i and v_j, and p is the number of triangles formed where one edge connects v_i and v_j.

3. Machine learning for prediction models

We randomly split the dataset into training and test datasets, and used the training dataset for developing the comorbidity index and fitting the model. Support Vector Machine (SVM) and eXtreme Gradient Boosting (XGBoost) algorithms were employed to construct prediction models that incorporated age and sex as additional covariates. We applied 10-fold cross-validation with grid-search on the training dataset to tune the hyperparameters in both SVM and XGBoost algorithms. Hyperparameters used for training SVM models： class_weight, 1:7; kernel, linear; C, 10; gamma, 0.1. Hyperparameters used for training XGBoost models: max_depth=6, learning_rate=0.05, n_estimators=100, booster='gbtree', gamma=0, min_child_weight=1, subsample=1, colsample_bytree=1, reg_alpha=0, reg_lambda=1. The Akaike information criterion (AIC) and Bayesian information criterion (BIC) as evaluation metrics were calculated using the following formulas [4,5]:

$$AIC = 2k + n\ln(MSE) \tag{8}$$

$$BIC = k\ln(\mathrm{n}) + n\ln(MSE) \tag{9}$$

Where k is the number of parameters in the fitted model, n is the number of observations, and MSE is mean square error.

References:

1. Hidalgo CA, Blumm N, Barabasi AL, Christakis NA: A dynamic network approach for the study of human phenotypes. PLoS Comput Biol 2009, 5(4): e1000353.
2. Katz D, Baptista J, Azen S, Pike M: Obtaining confidence intervals for the risk ratio in cohort studies. Biometrics 1978:469-474.
3. Jun Liu, Qingyu Xiong, Weiren Shi, Xin Shi, Kai Wang. Evaluating the importance of nodes in complex networks. Physica a-Statistical Mechanics and Its Applications 2016;452: 209-219.
4. Akaike H. Citation Classic - a New Look at the Statistical-Model Identification. Cc/Eng Tech Appl Sci 1981(51):22-22.
5.Andel J, Perez MG, Negrao AI. Estimating the Dimension of a Linear-Model. Kybernetika 1981;17(6):514-525.
