# Supplementary material for: Comorbidity patterns associated with severe COVID-19 outcomes: A cohort study based on the UK Biobank
Source: PLoS One. 2025 Aug 22;20(8):e0329701. doi: 10.1371/journal.pone.0329701 (PMC12373198; doi:10.1371/journal.pone.0329701)
Supplement: S1 Table — (PDF) [file pone.0329701.s002.pdf]

S1 Table. COVID-19 identification in primary care.

Code	Preferred term description
Y20d1	SARS-CoV-2 (severe acute respiratory syndrome coronavirus 2) RNA (ribonucleic acid) detection result positive
Y20cf	Suspected coronavirus disease 19 caused by severe acute respiratory syndrome coronavirus 2 (situation)
Y23ec	SARS-CoV-2 (severe acute respiratory syndrome coronavirus 2) IgG detection result positive
Y23f0	SARS-CoV-2 (severe acute respiratory syndrome coronavirus 2) IgM detection result positive
A795.	Coronavirus infection
A7y00	Coronavirus as cause of dis classified to other chapters
Y20fa	Coronavirus disease 19 caused by severe acute respiratory syndrome coronavirus 2 (disorder)
Y210b	Infection of upper respiratory tract caused by severe acute respiratory syndrome coronavirus 2 (disorder)
Y211c	Serotype 2019-nCoV (novel coronavirus)
Y228e	Coronavirus disease 19 caused by severe acute respiratory syndrome coronavirus 2 confirmed using clinical diagnostic criteria (situation)
Y20d1	SARS-CoV-2 (severe acute respiratory syndrome coronavirus 2) RNA (ribonucleic acid) detection result positive
Y20fb	Encephalopathy caused by severe acute respiratory syndrome coronavirus 2 (disorder)
Y20fc	Gastroenteritis caused by severe acute respiratory syndrome coronavirus 2 (disorder)
Y20fe	Myocarditis caused by severe acute respiratory syndrome coronavirus 2 (disorder)
Y20ff	Otitis media caused by severe acute respiratory syndrome coronavirus 2 (disorder)
Y210a	Pneumonia caused by severe acute respiratory syndrome coronavirus 2 (disorder)
Y23fd	Cardiomyopathy caused by severe acute respiratory syndrome coronavirus 2 (disorder)
XaaNq	Suspected coronavirus infection
Y20cf	Suspected coronavirus disease 19 caused by severe acute respiratory syndrome coronavirus 2 (situation)
Y22b7	Patient reported having already had Covid-19 in the recent past (not tested)
Y22b8	Patient reported having already had Covid-19 in the recent past (tested)
Y23f2	SARS-CoV-2 (severe acute respiratory syndrome coronavirus 2) total immunoglobulin arbitrary concentration in serum
Y244e	Arbitrary concentration of severe acute respiratory syndrome coronavirus 2 immunoglobulin A in serum (observable entity)
Y23f1	SARS-CoV-2 (severe acute respiratory syndrome coronavirus 2) IgM arbitrary concentration in serum
Y246f	Severe acute respiratory syndrome coronavirus 2 immunoglobulin A detected (finding)
Y23ed	SARS-CoV-2 (severe acute respiratory syndrome coronavirus 2) IgG arbitrary concentration in serum
Y212f	Antibody to 2019-nCoV (novel coronavirus)
Y23ec	SARS-CoV-2 (severe acute respiratory syndrome coronavirus 2) IgG detection result positive
Y23f0	SARS-CoV-2 (severe acute respiratory syndrome coronavirus 2) IgM detection result positive
Y240a	Severe acute respiratory syndrome coronavirus 2 immunoglobulin M qualitative existence in specimen (observable entity)
Y23f2	SARS-CoV-2 (severe acute respiratory syndrome coronavirus 2) total immunoglobulin arbitrary concentration in serum

Y23ed	SARS-CoV-2 (severe acute respiratory syndrome coronavirus 2) IgG arbitrary concentration in serum
Y23f1	SARS-CoV-2 (severe acute respiratory syndrome coronavirus 2) IgM arbitrary concentration in serum
Y244e	Arbitrary concentration of severe acute respiratory syndrome coronavirus 2 immunoglobulin A in serum (observable entity)
Y23ff	Severe acute respiratory syndrome coronavirus 2 immunoglobulin G qualitative existence in specimen (observable entity)
Y23e9	Has immunity to SARS-CoV-2 (severe acute respiratory syndrome coronavirus 2)
Y246f	Severe acute respiratory syndrome coronavirus 2 immunoglobulin A detected (finding)
Y210c	Severe acute respiratory syndrome coronavirus 2 serology (observable entity)
AyuDC	[X]Coronavirus infection, unspecified
X73lE	Coronavirus
X73lF	Human coronavirus
Y213a	Antigen of 2019-nCoV (novel coronavirus)
Y228d	Coronavirus disease 19 caused by severe acute respiratory syndrome coronavirus 2 confirmed by laboratory test (situation)
Y240b	SARS-CoV-2 (severe acute respiratory syndrome coronavirus 2) RNA (ribonucleic acid) qualitative existence in specimen
Y23f7	SARS-CoV-2 (severe acute respiratory syndrome coronavirus 2) detection result positive
441590008	Pneumonia caused by Severe acute respiratory syndrome coronavirus (disorder)
700217006	Suspected coronavirus infection
713084008	Pneumonia caused by Human coronavirus (disorder)
720294006	Severe acute respiratory syndrome-related coronavirus IgM
720293000	Immunoglobulin G antibody to Severe acute respiratory syndrome-related coronavirus
840533007	Severe acute respiratory syndrome coronavirus 2
840539006	Disease caused by 2019 novel coronavirus (disorder)
840544004	Suspected disease caused by 2019 novel coronavirus (situation)
840536004	Antigen of Severe acute respiratory syndrome coronavirus 2
866151004	Lymphocytopenia due to COVID-19
866152006	Thrombocytopenia due to 2019 novel coronavirus
870588003	Sepsis due to disease caused by COVID-19
870589006	Acute kidney injury due to disease caused by COVID-19
870590002	Acute hypoxemic respiratory failure due to disease caused by COVID-19
870591003	Rhabdomyolysis due to disease caused by Severe acute respiratory syndrome coronavirus 2 (disorder)
870362002	Immunoglobulin M antibody to Severe acute respiratory syndrome coronavirus 2
870361009	Immunoglobulin G antibody to Severe acute respiratory syndrome coronavirus 2
870362002	Severe acute respiratory syndrome coronavirus 2 IgM
897034005	Severe acute respiratory syndrome coronavirus 2 antibody test positive
1017214008	Severe acute respiratory syndrome coronavirus 2 viremia
1119302008	Acute COVID-19
1119303003	Post-acute COVID-19 (disorder)
1119304009	Chronic COVID-19 syndrome
1119343008	Severe acute respiratory syndrome coronavirus 2 mRNA
1300721000000109	Coronavirus disease 19 caused by severe acute respiratory syndrome coronavirus 2 confirmed by laboratory test

1321301000000101	Severe acute respiratory syndrome coronavirus 2 ribonucleic acid qualitative existence in specimen
1322781000000102	Severe acute respiratory syndrome coronavirus 2 antigen detection result positive
