# Supplementary material for: Comorbidity patterns associated with severe COVID-19 outcomes: A cohort study based on the UK Biobank
Source: PLoS One. 2025 Aug 22;20(8):e0329701. doi: 10.1371/journal.pone.0329701 (PMC12373198; doi:10.1371/journal.pone.0329701)
Supplement: S2 Table — (PDF) [file pone.0329701.s003.pdf]

S2 Table. The theoretical and observed infection rate in the study population.

Age group	No. of cases/population in England	Infection rate in England (per 100,000)	Population in the study	Theoretical no. of cases in the study population	Observed no. of cases in the study population
50-54	604,870/4,252,628	14220	15,978	2,273	2,063
55-59	501,982/4,195,969	11960	53,506	6,400	6,144
60-64	345,090/3,672,954	9400	60,544	5,692	5,553
65-69	222,614/3,025,304	7360	69,292	5,100	4,863
70-74	178,603/2,739,789	6520	92,427	6,027	5,096
75-79	130,252/2,406,517	5410	91,280	4,939	4,961
80+	197,093/2,434,531	8100	37,893	3,070	2,254
Total	2,180,504/22,727,692	9590	420,920	33,501	30,914
**Theoretical infection rate in the UKB participants: 9,590**					
**Observed infection rate in the UKB participants: 7,340**					
