# Supplementary material for: Comorbidity patterns associated with severe COVID-19 outcomes: A cohort study based on the UK Biobank
Source: PLoS One. 2025 Aug 22;20(8):e0329701. doi: 10.1371/journal.pone.0329701 (PMC12373198; doi:10.1371/journal.pone.0329701)
Supplement: S3 Table — (PDF) [file pone.0329701.s004.pdf]

S3 Table. List of 115 GBD codes used in the current study.

Disease name	Category	Corresponding ICD10 codes
HIV/AIDS	Infectious diseases	B20-B23.8, B24-B24.0, B97.81, C46-C46.52, C46.7-C46.9, F02.4
Sexually transmitted infections excluding HIV	Infectious diseases	A50-A60.9, A63-A64.0, B63, I98.0, K67.0-K67.2, M73.0-M73.8, N70-N71.9, N73-N74, N74.2-N74.8
Tuberculosis	Infectious diseases	A10-A14, A15-A18.89, A19-A19.9, B90-B90.9, K67.3, K93.0, M49.0, N74.0-N74.1
Lower respiratory infections	Infectious diseases	A48.1, A70, B96.0-B96.1, B97.21, B97.4-B97.6, J09-J18.2, J18.8-J18.9, J19.6-J22.9, J85.1, J91.0
Upper respiratory infections	Infectious diseases	J00-J06.9, J36-J36.0
Otitis media	Infectious diseases	H65-H70.93
Diarrheal disease	Infectious diseases	A00-A00.9, A02-A02.0, A02.8-A07, A07.2-A07.4, A08-A08.8, A09, K52.1
Typhoid and paratyphoid	Infectious diseases	A01-A01.4
Meningitis	Infectious diseases	A39-A39.9, A87-A87.9, D86.81, G00-G03.9, G06-G09.9
Encephalitis	Infectious diseases	A83-A85.2, A85.8-A86.0, B94.1, F07.1, G04-G05.8
Diphtheria	Infectious diseases	A36-A36.9
Whooping cough	Infectious diseases	A37-A37.91
Tetanus	Infectious diseases	A33-A35.0
Measles	Infectious diseases	B05-B05.9
Varicella and herpes zoster	Infectious diseases	B01-B02.9
Acute hepatitis	Infectious diseases	B15-B19.9, B94.2
Protein-energy malnutrition	Infectious diseases	E40-E46.9, E64.0
Iodine deficiency	Infectious diseases	E00-E02
Vitamin A deficiency	Infectious diseases	E50-E50.9, E64.1
Dietary iron deficiency	Infectious diseases	D50-D50.9
Lip and oral cavity cancer	Neoplasms	C00-C07, C08-C08.9
Nasopharynx cancer	Neoplasms	C11-C11.9
Esophageal cancer	Neoplasms	C15-C15.9
Stomach cancer	Neoplasms	C16-C16.9
Colon and rectum cancer	Neoplasms	C18-C19.0, C20, C21-C21.8
Liver cancer	Neoplasms	C22-C22.4, C22.7-C22.9
Gallbladder and biliary tract cancer	Neoplasms	C23, C24-C24.9
Pancreatic cancer	Neoplasms	C25-C25.9
Larynx cancer	Neoplasms	C32-C32.9
Tracheal, bronchus, and lung cancer	Neoplasms	C33, C34-C34.92
Malignant skin melanoma	Neoplasms	C43-C43.9
Non-melanoma skin cancer	Neoplasms	C44.01-C44.99
Breast cancer	Neoplasms	C50-C50.629, C50.8-C50.929
Cervical cancer	Neoplasms	C53-C53.9
Uterine cancer	Neoplasms	C54-C54.3, C54.8-C54.9

Ovarian cancer	Neoplasms	C56-C56.2, C56.9
Prostate cancer	Neoplasms	C61-C61.9
Testicular cancer	Neoplasms	C62-C62.92
Kidney cancer	Neoplasms	C64-C64.2, C64.9-C65.9
Bladder cancer	Neoplasms	C67-C67.9
Brain and central nervous diseases cancer	Neoplasms	C70-C70.1, C70.9-C72.9
Thyroid cancer	Neoplasms	C73
Mesothelioma	Neoplasms	C45-C45.2, C45.7, C45.9
Hodgkin lymphoma	Neoplasms	C81-C81.49, C81.7-C81.79, C81.9-C81.99
Non-Hodgkin lymphoma	Neoplasms	C82-C85.29, C85.7-C86.6, C96-C96.9
Multiple myeloma	Neoplasms	C88-C90.32
Leukemia	Neoplasms	C91-C93.7, C93.9-C95.2, C95.7-C95.92
Rheumatic heart disease	Circulatory system diseases	I01-I01.9, I02.0, I05-I09.9
Ischemic heart disease	Circulatory system diseases	I20-I21.6, I21.9-I25.9
Ischemic stroke	Circulatory system diseases	G45-G46.8, I63-I63.9, I65-I66.9, I67.2-I67.848, I69.3-I69.4
Intracerebral hemorrhage	Circulatory system diseases	I61-I62, I62.9, I69.0-I69.298
Subarachnoid hemorrhage	Circulatory system diseases	I60-I60.9, I67.0-I67.1
Hypertensive heart disease	Circulatory system diseases	I11-I11.2, I11.9
Non-rheumatic valvular heart diseases	Circulatory system diseases	I34-I37.9
Cardiomyopathy and myocarditis	Circulatory system diseases	B33.2-B33.20, B33.22-B33.24, D86.85, I40-I41.8, I42-I43.8, I51.4-I51.6
Atrial fibrillation and flutter	Circulatory system diseases	I48-I48.92
Peripheral artery disease	Circulatory system diseases	I70.2-I70.92, I73-I73.9
Endocarditis	Circulatory system diseases	B33.21, I33-I33.9, I38-I38.0, I39-I39.9
Chronic obstructive pulmonary disease	Respiratory system diseases	J41-J42.4, J43-J44.9
Pneumoconiosis	Respiratory system diseases	J60-J65.0, J92.0
Asthma	Respiratory system diseases	J45-J46.0
Interstitial lung diseases and pulmonary sarcoidosis	Respiratory system diseases	D86-D86.2, D86.9, J84-J84.9
Cirrhosis and other chronic liver disease	Digestive system diseases	I85-I85.9, I98.2, K70-K71, K71.3-K72, K72.1-K75, K75.2, K75.4-K76.2, K76.4-K77.8
Upper digestive disease	Digestive system diseases	K21-K21.9, K22.7-K22.719, K25-K30
Peptic ulcer disease	Digestive system diseases	K25-K28.9
Gastritis and duodenitis	Digestive system diseases	K29-K29.91
Gastroesophageal reflux diseases	Digestive system diseases	K21-K21.9,K22.7-K22.719
Appendicitis	Digestive system diseases	K35-K37.9
Paralytic ileus and intestinal obstruction	Digestive system diseases	K56-K56.9
Inguinal, femoral, and abdominal hernia	Digestive system diseases	K40-K42.9, K44-K46.9
Inflammatory bowel diseases	Digestive system diseases	K50-K51.319, K51.5-K52, K52.8-K52.9
Vascular intestinal disorders	Digestive system diseases	K55-K55.9
Gallbladder and biliary diseases	Digestive system diseases	K80-K80.81, K81-K83.9, K87-K87.1

Pancreatitis	Digestive system diseases	K85-K86.9
Alzheimer's diseases and other dementias	Neurological system diseases	F00-F02.0, F02.8-F03.91, F06.2, G30-G31.1, G31.8-G32.89
Parkinson's disease	Neurological system diseases	F02.3, G20-G20.9
Idiopathic epilepsy	Neurological system diseases	G40-G41.9
Multiple sclerosis	Neurological system diseases	G35-G35.0
Headache disorders	Neurological system diseases	G43-G44.89
Schizophrenia	Mental disorders	F20-F20.9, F25-F25.9
Depressive disorders	Mental disorders	F32-F33.9, F34.1
Bipolar disorder	Mental disorders	F30-F31.9, F34.0
Anxiety disorders	Mental disorders	F40-F44.9, F93-F93.2
Eating disorders	Mental disorders	F50-F50.9
Attention-deficit/hyperactivity disorder	Mental disorders	F90-F90.9
Conduct disorder	Mental disorders	F91-F92.9
Idiopathic developmental intellectual disability	Mental disorders	F70-F79.9
Alcohol use disorders	Mental disorders	E24.4, F10-F10.99, G31.2, G62.1
Drug use disorders	Mental disorders	F11-F19.99
Diabetes mellitus	Endocrine diseases	E08-E08.11, E08.3-E08.9, E10-E10.11, E10.3-E11.1, E11.3-E12.1, E12.3-E13.11, E13.3-E14.1, E14.3-E14.9
CKD (induced by DM)	Genitourinary system diseases	E10.2-E10.29, E11.2-E11.29
CKD (induced by HYPERTENSION)	Genitourinary system diseases	I12-I13.9
CKD	Genitourinary system diseases	N02-N08.8
Acute glomerulonephritis	Genitourinary system diseases	N00-N01.9
Dermatitis	Dermatologic condition	L20-L23.2, L23.4-L27, L27.2-L27.9, L30-L30.2, L30.5-L30.9
Psoriasis	Dermatologic condition	L40-L41.9
Bacterial skin disease	Dermatologic condition	A46-A46.0, A66-A67.3, A67.9, I89.1-I89.8, L00-L05.92, L08-L08.9, L30.3-L30.4, L88, L97-L98.499, M72.5-M72.6, N49.2-N49.3
Scabies	Dermatologic condition	B86
Fungal skin disease	Dermatologic condition	B35-B36.9
Viral skin disease	Dermatologic condition	B07-B09
Acne vulgaris	Dermatologic condition	L70-L70.9
Alopecia areata	Dermatologic condition	L63-L63.9
Pruritus	Dermatologic condition	L29-L29.9
Urticaria	Dermatologic condition	L50-L50.9
Decubitus ulcer	Dermatologic condition	L89-L89.95
Blindness and vision loss	Sensory system diseases	H25-H28.8, H31-H36.8, H40-H40.9, H42-H42.8, H46-H54.9
Glaucoma	Sensory system diseases	H40-H40.9, H42-H42.8
Cataract	Sensory system diseases	H25-H26.9, H28-H28.8
Age-related macular degeneration	Sensory system diseases	H35.3-H35.389
Refraction disorders	Sensory system diseases	H52-H52.7

Age-related and other hearing loss	Sensory system diseases	H71-H75.83, H80-H80.93, H83-H83.93, H90-H91, H91.1-H91.93, H94-H94.8
Rheumatoid arthritis	Musculoskeletal system diseases	M05-M05.9, M08-M09.8
Osteoarthritis	Musculoskeletal system diseases	M16-M18.9
Gout	Musculoskeletal system diseases	M10-M10.19, M10.3-M10.9, M1A00X0-M1A9XX1
Low back pain	Musculoskeletal system diseases	G54.4, M47.015-M47.019, M47.15-M47.18, M47.25-M47.28, M47.815-M47.818, M47.896-M47.899, M48.05-M48.08, M48.16-M48.19, M48.25-M48.27, M48.35-M48.38, M48.45-M48.48, M48.55-M48.58, M49.85-M49.88, M51.05-M51.07, M51.15-M51.17, M51.25-M51.27, M51.35-M51.37, M51.45-M51.47, M51.85-M51.87, M53.3, M53.85-M53.88, M54.05-M54.09, M54.15-M54.18, M54.3-M54.5, M99.03-M99.04, M99.13-M99.14, M99.23-M99.24, M99.33-M99.34, M99.43-M99.44, M99.53-M99.54, M99.63-M99.64, M99.73-M99.74, M99.83-M99.84
