# Supplementary material for: Comorbidity patterns associated with severe COVID-19 outcomes: A cohort study based on the UK Biobank
Source: PLoS One. 2025 Aug 22;20(8):e0329701. doi: 10.1371/journal.pone.0329701 (PMC12373198; doi:10.1371/journal.pone.0329701)
Supplement: S4 Table — (PDF) [file pone.0329701.s005.pdf]

S4 Table. Diagnostic codes for the identification of severe COVID-19.

Category	ICD-10 code	Disease name
**I. Certain infectious and parasitic diseases**	A00-A09	Intestinal infectious diseases
	A15-A19	Tuberculosis
	A20-A28	Certain zoonotic bacterial diseases
	A30-A49	Other bacterial diseases
	A50-A64	Infections with a predominantly sexual mode of transmission
	A65-A69	Other spirochetal diseases
	A70-A74	Other diseases caused by chlamydia
	A75-A79	Rickettsioses
	A80-A89	Viral infections of the central nervous system
	A92-A99	Arthropod-borne viral fevers and viral hemorrhagic fevers
	B00-B09	Viral infections characterized by skin and mucous membrane lesions
	B15-B19	Viral hepatitis
	B20-B24	Human immunodeficiency virus [HIV] disease
	B25-B34	Other viral diseases
	B35-B49	Mycoses
	B50-B64	Protozoal diseases
	B65-B83	Helminthiases
	B85-B89	Pediculosis, acariasis and other infestations
	B90-B94	Sequelae of infectious and parasitic diseases
	B95-B98	Bacterial, viral and other infectious agents
	B99-B99	Other infectious diseases
**II. Neoplasms**	C00-C97	Malignant neoplasms
	D00-D09	In situ neoplasms
	D10-D36	Benign neoplasms
	D37-D48	Neoplasms of uncertain or unknown behaviour
**III. Diseases of the blood and blood-forming organs and certain disorders involving the immune mechanism**	D50-D53	Nutritional anaemias
	D55-D59	Haemolytic anaemias
	D60-D64	Aplastic and other anaemias
	D65-D69	Coagulation defects, purpura and other haemorrhagic conditions
	D70-D77	Other diseases of blood and blood-forming organs
	D80-D89	Certain disorders involving the immune mechanism
**IV. Endocrine, nutritional and metabolic diseases**	E00-E07	Disorders of thyroid gland
	E10-E14	Diabetes mellitus
	E15-E16	Other disorders of glucose regulation and pancreatic internal secretion
	E20-E35	Disorders of other endocrine glands
	E40-E46	Malnutrition
	E50-E64	Other nutritional deficiencies
	E65-E68	Obesity and other hyperalimentation
	E70-E90	Metabolic disorders
**V. Mental and behavioral disorders**	F00-F09	Organic, including symptomatic, mental disorders

	F10-F19	Mental and behavioral disorders due to psychoactive substance use
	F20-F29	Schizophrenia, schizotypal and delusional disorders
	F30-F39	Mood [affective] disorders
	F40-F48	Neurotic, stress-related and somatoform disorders
	F50-F59	Behavioral syndromes associated with physiological disturbances and physical factors
	F60-F69	Disorders of adult personality and behavior
	F70-F79	Mental retardation
	F80-F89	Disorders of psychological development
	F90-F98	Behavioral and emotional disorders with onset usually occurring in childhood and adolescence
	F99-F99	Unspecified mental disorder
**VI. Diseases of the nervous system**	G00-G09	Inflammatory diseases of the central nervous system
	G10-G14	Systemic atrophies primarily affecting the central nervous system
	G20-G26	Extrapyramidal and movement disorders
	G30-G32	Other degenerative diseases of the nervous system
	G35-G37	Demyelinating diseases of the central nervous system
	G40-G47	Episodic and paroxysmal disorders
	G50-G59	Nerve, nerve root and plexus disorders
	G60-G64	Polyneuropathies and other disorders of the peripheral nervous system
	G70-G73	Diseases of myoneural junction and muscle
	G80-G83	Cerebral palsy and other paralytic syndromes
	G90-G99	Other disorders of the nervous system
**VII. Diseases of the eye and adnexa**	H00-H06	Disorders of eyelid, lacrimal system and orbit
	H10-H13	Disorders of conjunctiva
	H15-H22	Disorders of sclera, cornea, iris and ciliary body
	H25-H28	Disorders of lens
	H30-H36	Disorders of choroid and retina
	H40-H42	Glaucoma
	H43-H45	Disorders of vitreous body and globe
	H46-H48	Disorders of optic nerve and visual pathways
	H49-H52	Disorders of ocular muscles, binocular movement, accommodation and refraction
	H53-H54	Visual disturbances and blindness
	H55-H59	Other disorders of eye and adnexa
**VIII. Diseases of the ear and mastoid process**	H60-H62	Diseases of external ear
	H65-H75	Diseases of middle ear and mastoid
	H80-H83	Diseases of inner ear
	H90-H95	Other disorders of ear
**IX. Diseases of the circulatory system**	I00-I02	Acute rheumatic fever
	I05-I09	Chronic rheumatic heart diseases
	I10-I15	Hypertensive diseases
	I20-I25	Ischaemic heart diseases
	I26-I28	Pulmonary heart disease and diseases of pulmonary circulation

	I30-I52	Other forms of heart disease
	I60-I69	Cerebrovascular diseases
	I70-I79	Diseases of arteries, arterioles and capillaries
	I80-I89	Diseases of veins, lymphatic vessels and lymph nodes, not elsewhere classified
	I95-I99	Other and unspecified disorders of the circulatory system
**X. Diseases of the respiratory system**	J00-J06	Acute upper respiratory infections
	J09-J18	Influenza and pneumonia
	J20-J22	Other acute lower respiratory infections
	J30-J39	Other diseases of upper respiratory tract
	J40-J47	Chronic lower respiratory diseases
	J60-J70	Lung diseases due to external agents
	J80-J84	Other respiratory diseases principally affecting the interstitium
	J85-J86	Suppurative and necrotic conditions of lower respiratory tract
	J90-J94	Other diseases of pleura
	J95-J99	Other diseases of the respiratory system
**XI. Diseases of the digestive system**	K00-K14	Diseases of oral cavity, salivary glands and jaws
	K20-K31	Diseases of oesophagus, stomach and duodenum
	K35-K38	Diseases of appendix
	K40-K46	Hernia
	K50-K52	Noninfective enteritis and colitis
	K55-K64	Other diseases of intestines
	K65-K67	Diseases of peritoneum
	K70-K77	Diseases of liver
	K80-K87	Disorders of gallbladder, biliary tract and pancreas
	K90-K93	Other diseases of the digestive system
**XII. Diseases of the skin and subcutaneous tissue**	L00-L08	Infections of the skin and subcutaneous tissue
	L10-L14	Bullous disorders
	L20-L30	Dermatitis and eczema
	L40-L45	Papulosquamous disorders
	L50-L54	Urticaria and erythema
	L55-L59	Radiation-related disorders of the skin and subcutaneous tissue
	L60-L75	Disorders of skin appendages
	L80-L99	Other disorders of the skin and subcutaneous tissue
**XIII. Diseases of the musculoskeletal system and connective tissue**	M00-M25	Arthropathies
	M30-M36	Systemic connective tissue disorders
	M40-M54	Dorsopathies
	M60-M79	Soft tissue disorders
	M80-M94	Osteopathies and chondropathies

	M95-M99	Other disorders of the musculoskeletal system and connective tissue
**XIV. Diseases of the genitourinary system**	N00-N08	Glomerular diseases
	N10-N16	Renal tubulo-interstitial diseases
	N17-N19	Renal failure
	N20-N23	Urolithiasis
	N25-N29	Other disorders of kidney and ureter
	N30-N39	Other diseases of urinary system
	N40-N51	Diseases of male genital organs
	N60-N64	Disorders of breast
	N70-N77	Inflammatory diseases of female pelvic organs
	N80-N98	Noninflammatory disorders of female genital tract
	N99-N99	Other disorders of the genitourinary system
