# Supplementary material for: Comorbidity patterns associated with severe COVID-19 outcomes: A cohort study based on the UK Biobank
Source: PLoS One. 2025 Aug 22;20(8):e0329701. doi: 10.1371/journal.pone.0329701 (PMC12373198; doi:10.1371/journal.pone.0329701)
Supplement: S5 Table — (PDF) [file pone.0329701.s006.pdf]

S5 Table. COVID-19 vaccination identification in primary care.

Code	Preferred term description
Y210d	Severe acute respiratory syndrome coronavirus 2 vaccination (procedure)
Y211e	Severe acute respiratory syndrome coronavirus 2 vaccination declined (situation)
Y210d	Severe acute respiratory syndrome coronavirus 2 vaccination (procedure)
Y20fd	High priority for severe acute respiratory syndrome coronavirus 2 vaccination (finding)
840534001	COVID-19 vaccination
1119305005	COVID-19 antigen vaccine
1119349007	COVID-19 mRNA vaccine
1119350007	COVID-19 mRNA vaccination
1142180003	COVID-19 vaccine adverse reaction
1142181004	Adverse reaction to COVID-19 mRNA vaccine
1142182006	Adverse reaction to COVID-19 antigen vaccine
1144997007	First COVID-19 mRNA immunization
1144998002	Second COVID-19 mRNA immunization
1145003007	Hypersensitivity to COVID-19 mRNA vaccine
1145031003	COVID-19 vaccine declined
1145032005	COVID-19 mRNA vaccine declined
1145033000	COVID-19 antigen vaccine declined
1145034006	Second dose of COVID-19 mRNA vaccine declined
1156256003	COVID-19 vaccine adverse reaction
1156257007	COVID-19 immunization
1156270003	COVID-19 vaccine declined
1157106007	COVID-19 non-replicating viral vector vaccine adverse reaction
1157107003	COVID-19 non-replicating viral vector vaccination
1157108008	Second COVID-19 non-replicating viral vector vaccination
1157118003	COVID-19 non-replicating viral vector vaccine declined
1157120000	Second dose of COVID-19 non-replicating viral vector vaccine declined
1162643001	COVID-19 recombinant spike protein antigen vaccine
1162644007	COVID-19 recombinant spike protein antigen vaccine adverse reaction
1162645008	COVID-19 recombinant spike protein antigen vaccination
1162646009	Second dose of COVID-19 recombinant spike protein antigen vaccination
1162649002	COVID-19 recombinant spike protein antigen vaccine declined
1162650002	Second dose of COVID-19 recombinant spike protein antigen vaccine declined
1324681000000101	Administration of first dose of severe acute respiratory syndrome coronavirus 2 vaccine
1324721000000108	Severe acute respiratory syndrome coronavirus 2 vaccination dose declined
1324741000000101	Severe acute respiratory syndrome coronavirus 2 vaccination first dose declined
1324811000000107	Severe acute respiratory syndrome coronavirus 2 immunization course declined
1324691000000104	Administration of second dose of severe acute respiratory syndrome coronavirus 2 vaccine
1324751000000103	Severe acute respiratory syndrome coronavirus 2 vaccination second dose declined
1363791000000101	Administration of fourth dose of severe acute respiratory syndrome coronavirus 2 vaccine
1363831000000108	Administration of fifth dose of severe acute respiratory syndrome coronavirus 2 vaccine
1363861000000103	Administration of third dose of severe acute respiratory syndrome coronavirus 2 vaccine
1362651000000105	Severe acute respiratory syndrome coronavirus 2 protection maintenance course refused
1324721000000108	Severe acute respiratory syndrome coronavirus 2 vaccination dose declined
1324811000000107	Severe acute respiratory syndrome coronavirus 2 immunization course declined
