# Supplementary material for: Comorbidity patterns associated with severe COVID-19 outcomes: A cohort study based on the UK Biobank
Source: PLoS One. 2025 Aug 22;20(8):e0329701. doi: 10.1371/journal.pone.0329701 (PMC12373198; doi:10.1371/journal.pone.0329701)
Supplement: S6 Table — (PDF) [file pone.0329701.s007.pdf]

S6 Table. The ICD-10 codes for the identification of severe COVID-19 case.

ICD-10 code	Disease name	No. of cases
U07	COVID-19	2534
I20-I25	Ischemic heart diseases	149
E11-E14	Diabetes mellitus	132
J44-J47	Chronic lower respiratory diseases	128
I60-I69	Cerebrovascular diseases	94
I30-I52	Other forms of heart disease	77
F00-F09	Organic, including symptomatic, mental disorders	64
J09-J18	Influenza and pneumonia	57
I10-I15	Hypertensive diseases	53
K70-K77	Diseases of liver	44
N17-N19	Renal failure	42
C76-C80	Malignant neoplasms of ill-defined, secondary and unspecified sites	38
A30-A49	Other bacterial diseases	36
C15-C26	Malignant neoplasms of digestive organs	34
I70-I79	Diseases of arteries, arterioles and capillaries	32
E65-E68	Obesity and other hyperalimentation	32
G35-G37	Demyelinating diseases of the central nervous system	23
C69-C72	Malignant neoplasms of eye, brain and other parts of central nervous system	23
N30-N39	Other diseases of urinary system	22
G20-G26	Extrapyramidal and movement disorders	21
I26-I28	Pulmonary heart disease and diseases of pulmonary circulation	21
E00-E07	Disorders of thyroid gland	21
D37-D48	Neoplasms of uncertain or unknown behavior	18
J20-J22	Other acute lower respiratory infections	18
C50	Malignant neoplasm of breast	17
C73-C75	Malignant neoplasms of thyroid and other endocrine glands	17
C81-C96	Malignant neoplasms, stated or presumed to be primary, of lymphoid, hematopoietic and related tissue	15
C45-C49	Malignant neoplasms of mesothelial and soft tissue	15
J85-J86	Suppurative and necrotic conditions of lower respiratory tract	15
G30-G32	Other degenerative diseases of the nervous system	14
C30-C39	Malignant neoplasms of respiratory and intrathoracic organs	14
N10-N16	Renal tubulo-interstitial diseases	13
I80-I89	Diseases of veins, lymphatic vessels and lymph nodes, not elsewhere classified	12
D60-D64	Aplastic and other anemias	12
J80-J84	Other respiratory diseases principally affecting the interstitium	11
C00-C14	Malignant neoplasms of lip, oral cavity and pharynx	11
K20-K31	Diseases of esophagus, stomach and duodenum	10
C51-C58	Malignant neoplasms of female genital organs	9
C60-C63	Malignant neoplasms of male genital organs	9
D70-D77	Other diseases of blood and blood-forming organs	9
C64-C68	Malignant neoplasms of urinary tract	8
G10-G14	Systemic atrophies primarily affecting the central nervous system	8

C43-C44	Melanoma and other malignant neoplasms of skin	8
K80-K87	Disorders of gallbladder, biliary tract and pancreas	7
A80-A89	Viral infections of the central nervous system	7
G60-G64	Polyneuropathies and other disorders of the peripheral nervous system	7
K55-K64	Other diseases of intestines	5
J60-J69	Lung diseases due to external agents	2
J90-J94	Other diseases of pleura	1
K65-K67	Diseases of peritoneum	1
