# Supplementary material for: Comorbidity patterns associated with severe COVID-19 outcomes: A cohort study based on the UK Biobank
Source: PLoS One. 2025 Aug 22;20(8):e0329701. doi: 10.1371/journal.pone.0329701 (PMC12373198; doi:10.1371/journal.pone.0329701)
Supplement: S7 Table — Adjusted for age, sex, annual household income, TDI, BMI, smoking status, drinking status, and the disease status of other modules. (PDF) [file pone.0329701.s008.pdf]

**S7 Table. The association between each individual disease and the risk of severe COVID-19.**

| Disease name | Subordinate disease module | OR[a] | 95%CI |
|---|---|---|---|
| Glaucoma | Sensory disease module | 1.180 | 0.955-1.451 |
| Cataract | Sensory disease module | 1.478 | 1.295-1.684 |
| Age-related macular degeneration | Sensory disease module | 1.243 | 0.935-1.641 |
| Refraction disorders | Sensory disease module | 1.210 | 0.877-1.649 |
| Dietary iron deficiency | Digestive disease module | 1.351 | 1.134-1.606 |
| Cirrhosis and other chronic liver diseases | Digestive disease module | 1.681 | 1.383-2.038 |
| Gastritis and duodenitis | Digestive disease module | 1.102 | 0.975-1.244 |
| Gastroesophageal reflux disease | Digestive disease module | 1.123 | 0.999-1.261 |
| Inguinal, femoral, and abdominal hernia | Digestive disease module | 1.212 | 1.089-1.347 |
| Gallbladder and biliary diseases | Digestive disease module | 1.308 | 1.093-1.561 |
| Diabetes mellitus | Cardiometabolic disease module | 1.919 | 1.627-2.259 |
| CKD (induced by DM) | Cardiometabolic disease module | 1.526 | 1.313-1.771 |
| CKD (induced by hypertension) | Cardiometabolic disease module | 1.279 | 1.139-1.434 |
| Rheumatoid arthritis | Cardiometabolic disease module | 1.064 | 0.809-1.388 |
| Osteoarthritis | Cardiometabolic disease module | 0.912 | 0.816-1.019 |
| Low back pain | Cardiometabolic disease module | 0.937 | 0.845-1.038 |
| Gout | Cardiometabolic disease module | 1.123 | 0.982-1.282 |
| Hypertensive heart disease | Cardiometabolic disease module | 1.224 | 1.091-1.372 |
| Atrial fibrillation and flutter | Circulatory and respiratory disease module | 1.932 | 1.682-2.217 |
| Peripheral artery disease | Circulatory and respiratory disease module | 1.523 | 1.264-1.831 |
| Ischemic heart disease | Circulatory and respiratory disease module | 1.271 | 1.142-1.413 |
| Ischemic stroke | Circulatory and respiratory disease module | 1.897 | 1.624-2.214 |
| Non-rheumatic valvular heart disease | Circulatory and respiratory disease module | 1.492 | 1.215-1.827 |
| Chronic obstructive pulmonary disease | Circulatory and respiratory disease module | 1.585 | 1.376-1.824 |
| Pneumoconiosis | Circulatory and respiratory disease module | 1.099 | 0.811-1.478 |
| Asthma | Circulatory and respiratory disease module | 1.201 | 1.067-1.35 |
| Rheumatic heart disease | Circulatory and respiratory disease module | 1.438 | 1.160-1.777 |
| Decubitus ulcer | Intestinal disease module | 2.473 | 1.807-3.391 |
| Diarrheal diseases | Intestinal disease module | 1.017 | 0.896-1.151 |
| Diphtheria | Intestinal disease module | 0.933 | 0.787-1.103 |
| Inflammatory bowel disease | Intestinal disease module | 1.197 | 1.024-1.395 |
| Lower respiratory infections | Intestinal disease module | 1.175 | 1.059-1.303 |
| Depressive disorders | Psychological related disease module | 1.388 | 1.213-1.586 |
| Alcohol use disorders | Psychological related disease module | 1.467 | 1.226-1.751 |
| Drug use disorders | Psychological related disease module | 1.367 | 1.185-1.575 |
| Dermatitis | Psychological related disease module | 1.048 | 0.937-1.171 |
| Urticaria | Psychological related disease module | 1.073 | 0.853-1.340 |

| | | | |
|---|---|---|---|
| Psoriasis | Psychological related disease module | 1.137 | 0.924-1.392 |
| Fungal skin diseases | Psychological related disease module | 0.854 | 0.746-0.976 |
| Pruritus | Psychological related disease module | 0.901 | 0.737-1.095 |
| Age-related and other hearing loss | Infectious disease module | 0.982 | 0.866-1.111 |
| Typhoid and paratyphoid | Infectious disease module | 0.846 | 0.630-1.122 |
| Bacterial skin diseases | Infectious disease module | 0.974 | 0.879-1.078 |
| Anxiety disorders | Infectious disease module | 0.996 | 0.893-1.110 |
| Idiopathic epilepsy | Infectious disease module | 1.213 | 1.029-1.428 |
| Headache disorders | Infectious disease module | 0.939 | 0.814-1.082 |
| Viral skin diseases | Infectious disease module | 1.012 | 0.832-1.227 |
| Sexually transmitted infections excluding HIV | Infectious disease module | 0.877 | 0.765-1.003 |
| Tuberculosis | Infectious disease module | 0.848 | 0.720-0.995 |
| Upper respiratory infections | Infectious disease module | 0.911 | 0.822-1.010T |
| Otitis media | Infectious disease module | 0.894 | 0.772-1.034 |

[a]Adjusted for age, sex, annual household income, TDI, BMI, smoking status, drinking status, and the disease status of other modules
