# Supplementary material for: Comorbidity patterns associated with severe COVID-19 outcomes: A cohort study based on the UK Biobank
Source: PLoS One. 2025 Aug 22;20(8):e0329701. doi: 10.1371/journal.pone.0329701 (PMC12373198; doi:10.1371/journal.pone.0329701)
Supplement: S8 Table — (PDF) [file pone.0329701.s009.pdf]

S8 Table. The node importance of diseases in seven comorbidity modules.

Disease	Module	Node importance
Cataract	Age-related eye disease module	5.00
Diphtheria	Gastrointestinal disease module	3.39
Asthma	Circulatory and respiratory disease module	3.14
Atrial fibrillation and flutter	Circulatory and respiratory disease module	2.50
Ischemic heart disease	Circulatory and respiratory disease module	2.48
Chronic obstructive pulmonary disease	Circulatory and respiratory disease module	2.25
Lower respiratory infections	Gastrointestinal disease module	1.93
Rheumatic heart disease	Circulatory and respiratory disease module	1.72
Non-rheumatic valvular heart disease	Circulatory and respiratory disease module	1.67
Age-related macular degeneration	Age-related eye disease module	1.67
Refraction disorders	Age-related eye disease module	1.67
Glaucoma	Age-related eye disease module	1.67
Gallbladder and biliary diseases	Digestive disease module	1.60
Inguinal, femoral, and abdominal hernia	Digestive disease module	1.60
Gastroesophageal reflux disease	Digestive disease module	1.60
Gastritis and duodenitis	Digestive disease module	1.60
Dermatitis	Mental and skin disorder module	1.58
Rheumatoid arthritis	Cardiometabolic disease module	1.56
Pruritus	Mental and skin disorder module	1.51
Inflammatory bowel disease	Gastrointestinal disease module	1.38
Depressive disorders	Mental and skin disorder module	1.35
Gout	Cardiometabolic disease module	1.29
Cirrhosis and other chronic liver diseases	Digestive disease module	1.28
Dietary iron deficiency	Digestive disease module	1.28
Hypertensive heart disease	Cardiometabolic disease module	1.22
CKD (induced by HYPERTENSION)	Cardiometabolic disease module	1.21
Ischemic stroke	Circulatory and respiratory disease module	0.99
CKD (induced by DM)	Cardiometabolic disease module	0.84
Diarrheal diseases	Gastrointestinal disease module	0.83
Alcohol use disorders	Mental and skin disorder module	0.67
Drug use disorders	Mental and skin disorder module	0.67
Fungal skin diseases	Mental and skin disorder module	0.67
Diabetes mellitus	Cardiometabolic disease module	0.60
Decubitus ulcer	Gastrointestinal disease module	0.42
Osteoarthritis	Cardiometabolic disease module	0.40
Viral skin diseases	Infectious and neuropsychiatric disease module	0.37
Psoriasis	Mental and skin disorder module	0.34
Urticaria	Mental and skin disorder module	0.34
Peripheral artery disease	Circulatory and respiratory disease module	0.33
Pneumoconiosis	Circulatory and respiratory disease module	0.33
Sexually transmitted infections excluding HIV	Infectious and neuropsychiatric disease module	0.32
Tuberculosis	Infectious and neuropsychiatric disease module	0.32

Age-related and other hearing loss	Infectious and neuropsychiatric disease module	0.30
Anxiety disorders	Infectious and neuropsychiatric disease module	0.30
Idiopathic epilepsy	Infectious and neuropsychiatric disease module	0.27
Headache disorders	Infectious and neuropsychiatric disease module	0.22
Low back pain	Cardiometabolic disease module	0.20
Typhoid and paratyphoid	Infectious and neuropsychiatric disease module	0.15
Upper respiratory infections	Infectious and neuropsychiatric disease module	0.07
Bacterial skin diseases	Infectious and neuropsychiatric disease module	0.03
Otitis media	Infectious and neuropsychiatric disease module	0.03
