# Supplementary material for: Comorbidity patterns associated with severe COVID-19 outcomes: A cohort study based on the UK Biobank
Source: PLoS One. 2025 Aug 22;20(8):e0329701. doi: 10.1371/journal.pone.0329701 (PMC12373198; doi:10.1371/journal.pone.0329701)
Supplement: S9 Table — AIC, Akaike Information Criterion; AUC, the area under the receiver operating characteristic curve; BIC, Bayesian Information Criterion; CCI, Charlson Comorbidity Index; SVM, Support Vector Machine; XGBoost, EXtreme Gradient Boosting.16-comorbidity based index was reported in previous literature, including 16 diseases. The full module-based comorbidity index included all 51 diseases. (PDF) [file pone.0329701.s010.pdf]

S9 Table. Comparison of comorbidity indices in predicting risk of severe COVID-19.

Algorithm	Comorbidity index	Primary evaluation metric		Secondary evaluation metric	
		AUC		AIC	BIC
XGBoost	CCI	0.714		-14131	-14111
	16-comorbidity based index	0.714		-14060	-13989
	Full module-based comorbidity index	0.779		-14531	-14511
	Simplified module-based comorbidity indices (No. of diseases for calculating comorbidity index)				
	40	0.775		-14515	-14495
	35	0.778		-14565	-14545
	30	0.772		-14498	-14478
	25	0.774		-14502	-14482
	20	0.763		-14457	-14437
	15	0.762		-14409	-14381
SVM	CCI	0.679		-6197	-6177
	16-comorbidity based index	0.678		-6089	-5967
	Full module-based comorbidity index	0.710		-7675	-7655
	Simplified module-based comorbidity indices (No. of diseases for calculating comorbidity index)				
	40	0.709		-7623	-7603
	35	0.708		-7696	-7676
	30	0.707		-7576	-7555
	25	0.704		-7531	-7511
	20	0.703		-7299	-7279
	15	0.701		-7183	-7162

AIC, Akaike Information Criterion; AUC, the area under the receiver operating characteristic curve; BIC, Bayesian Information Criterion; CCI, Charlson Comorbidity Index; SVM, Support Vector Machine; XGBoost, EXtreme Gradient Boosting.16-comorbidity based index was reported in previous literature, including 16 diseases. The full module-based comorbidity index included all 51 diseases.
