# Supplementary figures and images for: Comorbidity patterns associated with severe COVID-19 outcomes: A cohort study based on the UK Biobank
Source: PLoS One. 2025 Aug 22;20(8):e0329701. doi: 10.1371/journal.pone.0329701 (PMC12373198; doi:10.1371/journal.pone.0329701)

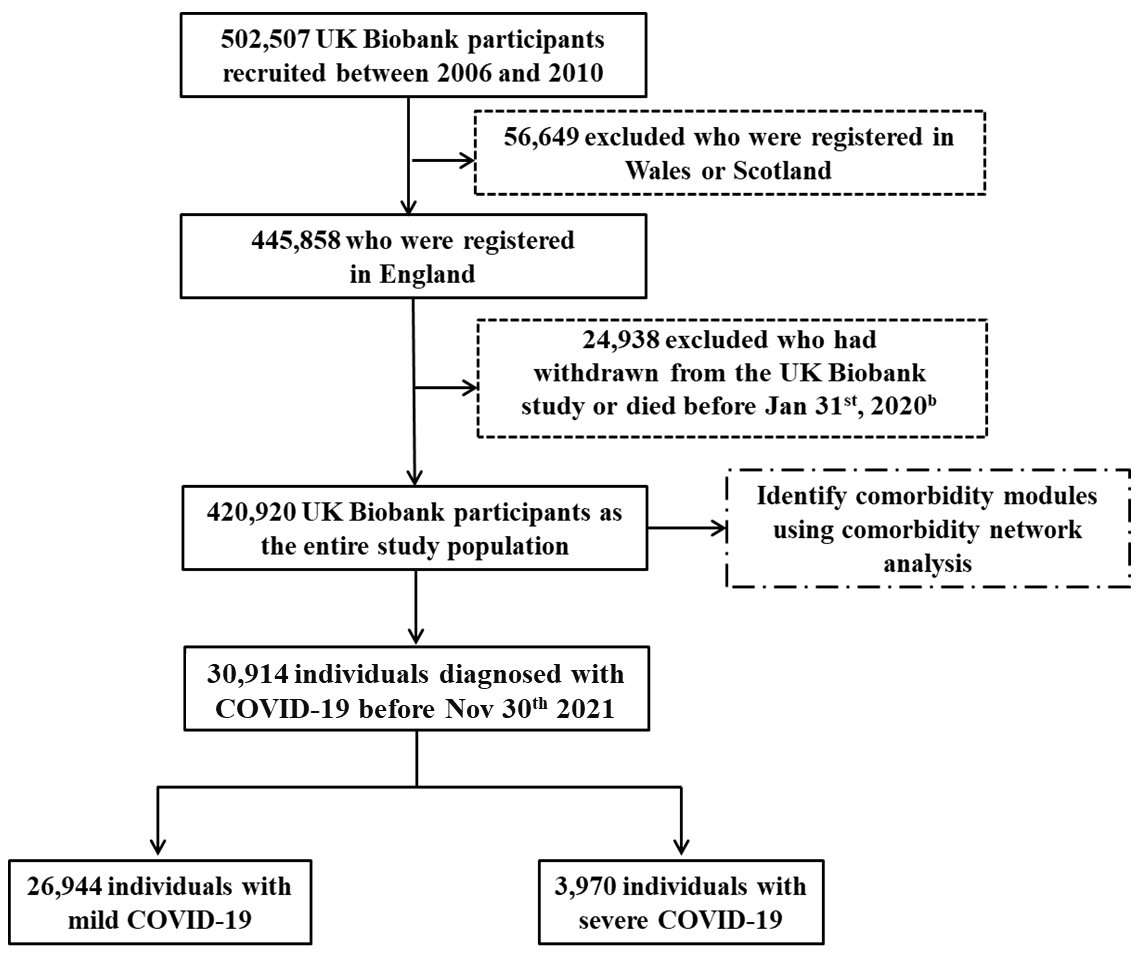

Supplement: S1 Fig — The first COVID-19 case was confirmed on 31 January 2020 in the UK. (TIF) [file pone.0329701.s011.tif]

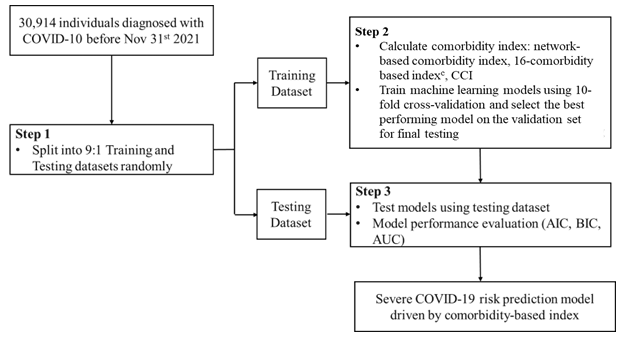

Supplement: S2 Fig — AIC, Akaike Information Criterion; AUC, the area under the receiver operating characteristic curve; BIC, Bayesian Information Criterion; CCI, Charlson Comorbidity Index. c16 comorbidities reported in previous literature, including coronary artery disease, heart failure, atrial fibrillation, T1DM, T2DM, hypertension, asthma, COPD, cancer, dementia, depression, anxiety, psychosis, bipolar, cognitive impairment, and stroke. (TIF) [file pone.0329701.s012.tif]

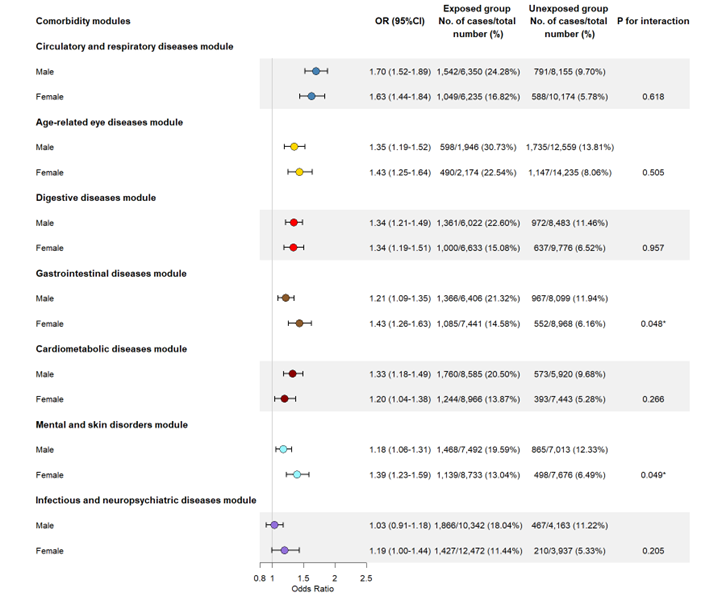

Supplement: S3 Fig — ORs (95%CI) were derived from fully adjusted logistic models (adjusted for age, Townsend deprivation index, annual household income, BMI, smoking status, drinking status, and disease status of other modules). The interaction between comorbidity modules and sex was evaluated by including the interaction term in the logistic models. * P < .05. (TIF) [file pone.0329701.s013.tif]

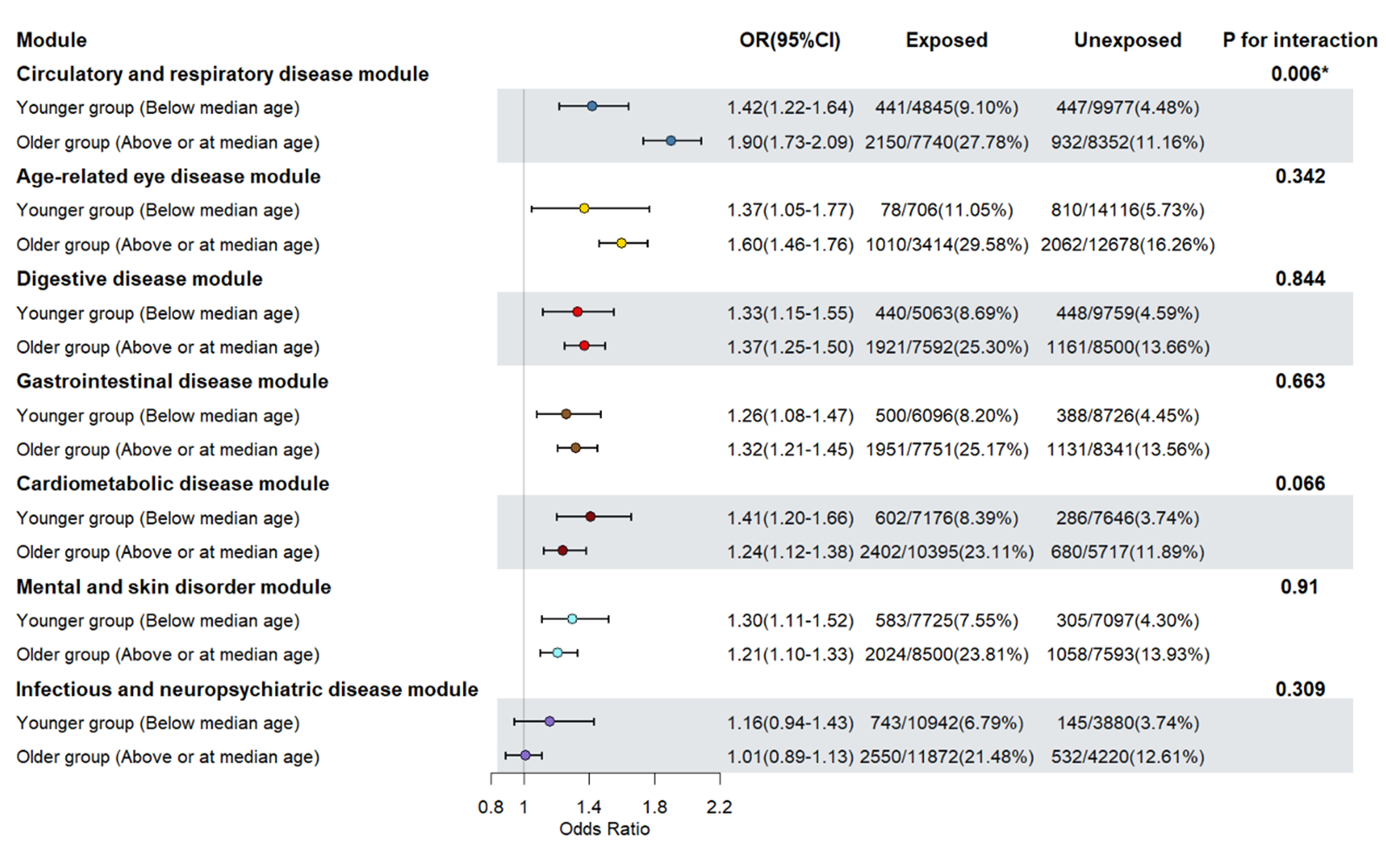

Supplement: S4 Fig — ORs (95%CI) were derived from fully adjusted logistic models (adjusted for sex, Townsend deprivation index, annual household income, BMI, smoking status, drinking status, and disease status of other modules). The interaction between comorbidity modules and sex was evaluated by including the interaction term in the logistic models. * P < .05. (TIF) [file pone.0329701.s014.tif]

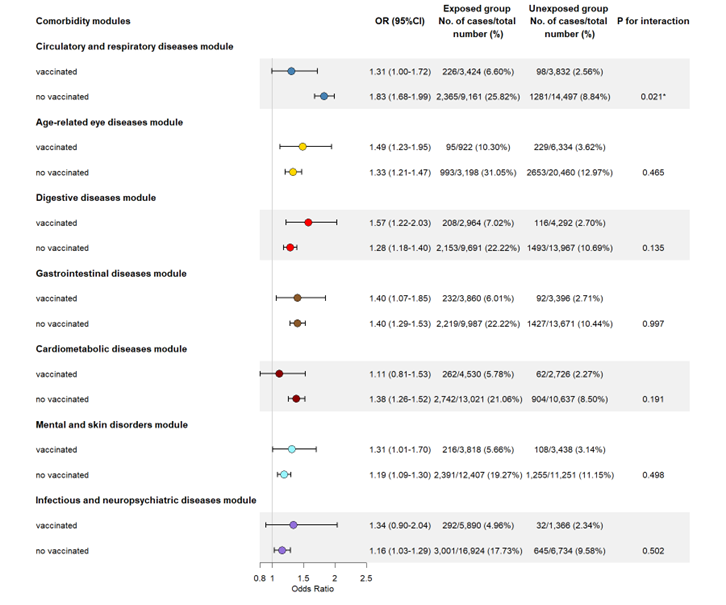

Supplement: S5 Fig — ORs (95%CI) were derived from fully adjusted logistic models (adjusted for age, sex, Townsend deprivation index, annual household income, BMI, smoking status, drinking status, and disease status of other modules). The interaction between comorbidity modules and vaccination status was evaluated by including the interaction term in the logistic models. * P < .05. (TIF) [file pone.0329701.s015.tif]

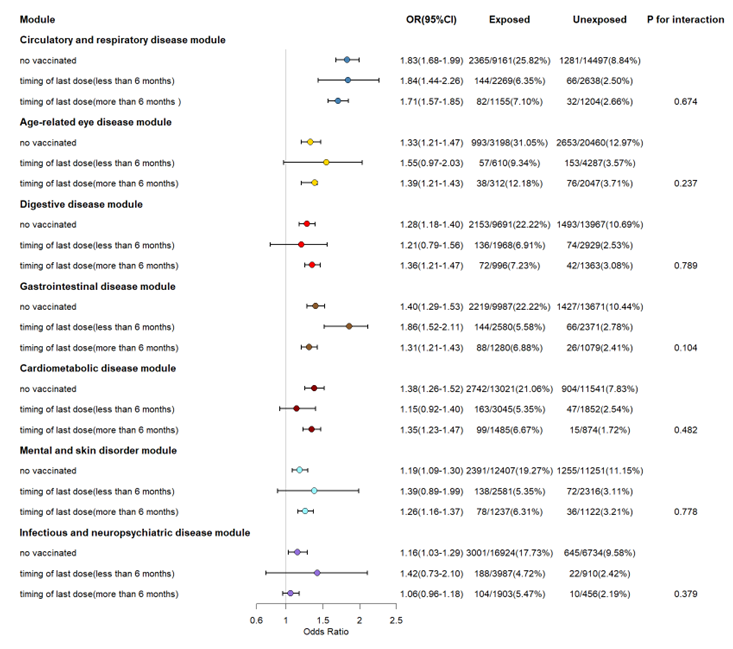

Supplement: S6 Fig — ORs (95%CI) were derived from fully adjusted logistic models (adjusted for age, sex, Townsend deprivation index, annual household income, BMI, smoking status, drinking status, and disease status of other modules). (TIF) [file pone.0329701.s016.tif]

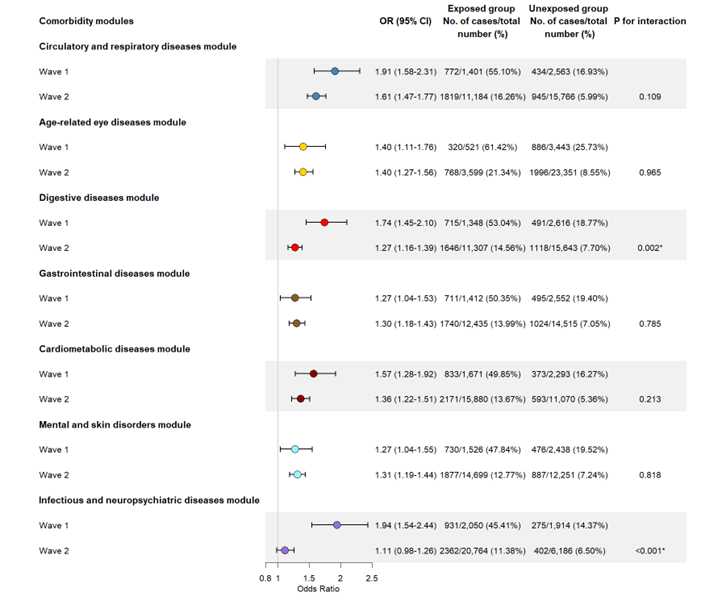

Supplement: S7 Fig — ORs (95%CI) were derived from fully adjusted logistic models (adjusted for age, sex, Townsend deprivation index, annual household income, BMI, smoking status, drinking status, and disease status of other modules). The interaction between comorbidity modules and different waves was evaluated by including the interaction term in the logistic models. * P < .05. (TIF) [file pone.0329701.s017.tif]
